# Comparative effects of weight-loss diet, exercise training, respiratory muscle training, and oropharyngeal muscle training in obstructive sleep apnea: a systematic review and network meta-analysis

**DOI:** 10.3389/fmed.2026.1789371

**Published:** 2026-03-19

**Authors:** Xiang Lin, Dongqiang Liu, Wen Sun, Wenshan Xu, Anqi Wang, Jiazhe Lv, Caixia Qiu, Shungui Xu

**Affiliations:** 1 Affiliated People’s Hospital of Fujian University of Traditional Chinese Medicine, Fuzhou, Fujian, China; 2 Fujian University of Traditional Chinese Medicine, Fuzhou, Fujian, China; 3 School of Mathematics and Statistics, Fuzhou University, Fuzhou, Fujian, China

**Keywords:** exercise training, obstructive sleep apnea, oropharyngeal muscle training, respiratory muscle training, weight-loss diet

## Abstract

**Background:**

Obstructive sleep apnea (OSA) is a common sleep disorder and has become a significant public health issue. Weight-loss diet (WLD), exercise training (ET), respiratory muscle training (RMT), and oropharyngeal muscle training (OMT) have been shown to improve OSA symptoms to some extent, offering new treatment options.

**Objective:**

To compare the relative efficacy of WLD, ET, RMT, and OMT in patients with OSA through a systematic review and network meta-analysis, and to provide evidence-based support for clinical decision-making on lifestyle and functional training interventions in OSA management.

**Methods:**

PubMed, EMBASE, Web of Science, and the Cochrane Library were systematically searched from inception to December 2025, with an updated search performed in January 2026. The primary outcome was the apnea-hypopnea index (AHI), while secondary outcomes included the Epworth Sleepiness Scale (ESS), Pittsburgh Sleep Quality Index (PSQI), and body mass index (BMI). Risk of bias was assessed using RevMan 5.4, and the GRADE method was used to evaluate the quality of evidence for all outcomes. Network meta-analysis was conducted using the “network” command in STATA 17.0.

**Results:**

ET exhibited the most significant efficacy in reducing the AHI (MD = −9.13, 95% CI: −12.04 to −6.21, SUCRA = 89.9%) and the PSQI (MD = −2.20, 95% CI: −3.23 to −1.16, SUCRA = 78.1%); OMT yielded the greatest reduction in the ESS score (MD = −4.00, 95% CI: −5.45 to −2.56, SUCRA = 92.2%); WLD showed the most prominent effect in reducing the BMI (MD = −2.39, 95% CI: −3.95 to −0.84, SUCRA = 96.2%).

**Conclusion:**

Lifestyle and functional training interventions demonstrate distinct outcome-specific effects in the management of OSA. These findings suggest that individualized intervention strategies should be selected in clinical practice based on patients’ predominant symptoms and therapeutic goals. However, the effects of these interventions still require further high-quality evidence to be fully validated.

## 1 Introduction

Obstructive sleep apnea (OSA) is a common sleep-related breathing disorder characterized by recurrent upper airway collapse during sleep, resulting in intermittent hypopnea or apnea. These events lead to chronic intermittent hypoxia, sleep fragmentation, and sustained sympathetic activation (1, 2). OSA is a systemic disorder that affects multiple organ systems and is strongly associated with an increased risk of cardiovascular, metabolic, and neurological conditions, including hypertension, diabetes mellitus, and heart failure (3, 4). In addition, excessive daytime sleepiness and neurocognitive impairment associated with OSA substantially reduce quality of life and increase the risk of adverse outcomes such as traffic accidents and occupational injuries (5). Epidemiological evidence suggests that OSA represents a major global health burden. It is estimated that approximately 936 million adults aged 30–69 years worldwide are affected by OSA, of whom about 425 million have moderate-to-severe disease (6). Given the limited public awareness of OSA and its relatively low diagnostic rate, the true prevalence is likely underestimated. Therefore, identifying safe, effective, and sustainable intervention strategies for long-term OSA management is of considerable clinical and public health importance.

Current treatment options for OSA include continuous positive airway pressure (CPAP), oral appliances, and surgical interventions (7). CPAP is widely recognized as the first-line therapy for moderate-to-severe OSA; however, its long-term effectiveness is often limited by poor adherence and suboptimal tolerance (8). Oral appliances and surgical treatments may benefit selected patients, but their applicability is constrained by narrow indications, interindividual variability in treatment response, or the invasive nature of surgical procedures (9). As a result, non-device-based conservative interventions centered on lifestyle modification and functional training have received increasing attention and are now considered important components of comprehensive OSA management. These conservative approaches primarily include weight-loss diet (WLD), exercise training (ET), respiratory muscle training (RMT), and oropharyngeal muscle training (OMT). Previous studies have shown that these interventions may exert beneficial effects on key OSA-related outcomes, such as reducing the apnea-hypopnea index (AHI), alleviating daytime sleepiness, and improving sleep quality (10–13). However, direct comparisons of the relative efficacy among these interventions remain limited. Although existing studies have compared the efficacy of ET, RMT, and OMT, no study has directly compared the efficacy of WLD with the above three interventions (14). In addition, WLD is often grouped into a single category of lifestyle interventions for general analysis, without directly comparing its independent efficacy with other functional training modalities (15). However, as a core lifestyle intervention for OSA, the clinical value of WLD has been explicitly recognized by multiple clinical guidelines (16, 17).

Therefore, the present study aims to systematically and for the first time compare the effects of WLD, ET, RMT, and OMT on major clinical outcomes in patients with OSA using a network meta-analysis framework. By integrating direct and indirect evidence and applying probabilistic ranking methods, this study seeks to clarify the relative effectiveness of different lifestyle and functional training interventions, thereby providing evidence-based guidance for individualized clinical decision-making in the management of OSA.

## 2 Methods

### 2.1 Protocol and reporting standards

This systematic review and network meta-analysis was prospectively registered in the International Prospective Register of Systematic Reviews (PROSPERO; registration number: CRD420251013124). The study was conducted in accordance with the Cochrane Handbook for Systematic Reviews of Interventions and reported following the Preferred Reporting Items for Systematic Reviews and Meta-Analyses for Network Meta-Analyses (PRISMA-NMA) guidelines (18).

### 2.2 Search strategy

A comprehensive and systematic literature search was performed in PubMed, EMBASE, Web of Science, and the Cochrane Library from database inception to December 2025, with an updated search performed in January 2026. Search terms were constructed using a combination of Medical Subject Headings and free-text keywords related to obstructive sleep apnea and non-pharmacological interventions, including but not limited to “Sleep Apnea, Obstructive,” “Exercise,” and “Diet.” Relevant synonyms and expanded terms were also incorporated to maximize search sensitivity. The detailed search strategy for each database is provided in Appendix 1.

### 2.3 Inclusion criteria

Eligibility criteria were defined according to the PICOS framework as follows:

(1) Participants: Adults (≥18 years), of either sex, with OSA diagnosed by polysomnography (PSG), defined by an AHI ≥ 5 events/h. (2) Interventions: Participants in the intervention groups received one of the following interventions: a WLD, ET, RMT, or OMT. WLD was defined as a structured dietary intervention primarily focused on caloric restriction with the aim of body weight reduction. ET was defined as a structured aerobic and/or resistance exercise program designed to improve cardiorespiratory fitness and/or skeletal muscle function. RMT referred to a systematic load-based intervention targeting the respiratory muscles using specific devices, such as threshold pressure trainers. OMT was defined as a structured training program aimed at improving the function of upper airway dilator muscles, including the tongue muscles, soft palate muscles, and pharyngeal muscles. (3) Comparators: The control group (CG) received health education, no intervention, sham intervention, or one of the four interventions described above. (4) Outcomes: Eligible studies reported at least one of the following outcomes: AHI, Epworth Sleepiness Scale (ESS), Pittsburgh Sleep Quality Index (PSQI), or body mass index (BMI). (5) Study design: Only randomized controlled trials (RCTs) were included, with no restrictions on publication language.

### 2.4 Exclusion criteria

Studies were excluded if they met any of the following criteria:

(1) Use of combination therapies, such as concurrent CPAP, mandibular advancement device (MAD), surgical interventions, or pharmacological treatments. (2) Review articles, conference abstracts, or academic theses. (3) Case reports, animal studies, or study protocols. (4) Studies with missing outcome data or results that could not be extracted. (5) Duplicate publications or studies with clearly overlapping data. (6) Studies for which the full text was unavailable.

### 2.5 Study selection and data extraction

All records retrieved from the database searches were imported into EndNote X9 for reference management, and duplicate records were removed. Two investigators independently screened the titles and abstracts of the remaining records according to the predefined inclusion and exclusion criteria. Full texts of potentially eligible studies were then obtained and independently assessed in detail, with reasons for exclusion recorded. Extracted information included the first author, year of publication, country of study, sample size, participant age, baseline AHI and BMI for both intervention and control groups, intervention characteristics (type, frequency, and duration), comparator interventions, and primary/secondary outcome measures. Any discrepancies during the screening or data extraction process were resolved through discussion; if consensus could not be reached, a third investigator was consulted to make the final decision, ensuring the objectivity and reproducibility of the study process. For studies that reported only pre- and post-intervention data or presented outcomes in alternative formats (e.g., medians and interquartile ranges), data were converted in accordance with the Cochrane Handbook for Systematic Reviews of Interventions to calculate mean changes and their corresponding standard deviations (19), as detailed in Appendix 2.

### 2.6 Assessment of risk of bias and quality of evidence

The methodological quality of the included RCTs was assessed using the Cochrane Risk of Bias tool (RoB 1) (20). The assessment covered seven potential domains of bias: random sequence generation, allocation concealment, blinding of participants and personnel, blinding of outcome assessment, completeness of outcome data, selective outcome reporting, and other sources of bias. Each domain was independently judged as having a low, high, or unclear risk of bias. Risk of bias assessment was performed independently by two investigators. Any disagreements were resolved through discussion, and if consensus could not be reached, a third investigator was consulted until agreement was achieved.

This study employed the Grading of Recommendations Assessment, Development and Evaluation (GRADE) method to assess the quality of evidence for each outcome. The assessment considered five downgrading factors: risk of bias, inconsistency, indirectness, imprecision, and publication bias (21). Based on these factors, the quality of evidence was categorized into four levels: very low, low, moderate, and high.

### 2.7 Statistical analysis

RevMan 5.4 was used to assess the risk of bias of the included studies, and both conventional meta-analyses and network meta-analysis were performed using STATA version 17.0. For continuous outcomes, including AHI, ESS, PSQI, and BMI, mean changes with corresponding standard deviations before and after the intervention were analyzed. Since all studies employed consistent measurement scales, effect sizes were expressed as mean differences (MDs) with 95% confidence intervals (CIs). Heterogeneity was quantified using the *I*^2^ statistic, with an *I*^2^ value greater than 50.00% indicating significant heterogeneity. When *p* ≥ 0.05 and *I*^2^ ≤ 50.00%, a fixed-effect model was used; otherwise, a random-effects model was applied. Network plots, forest plots, and funnel plots were generated, and relevant statistical parameters were calculated. To assess the consistency of the network model, local inconsistency was evaluated using the node-splitting approach when closed loops were present, combined with loop inconsistency tests to examine the agreement between direct and indirect evidence within closed loops; when no closed loops were formed, a consistency model was directly applied (22). On this basis, the surface under the cumulative ranking curve (SUCRA) was used to rank the relative efficacy of different interventions. Sensitivity analysis was performed to evaluate the robustness of the results. Finally, publication bias and small-study effects were assessed by funnel plot symmetry and further evaluated using Egger’s test.

## 3 Results

### 3.1 Selection process

**Figure 1:**
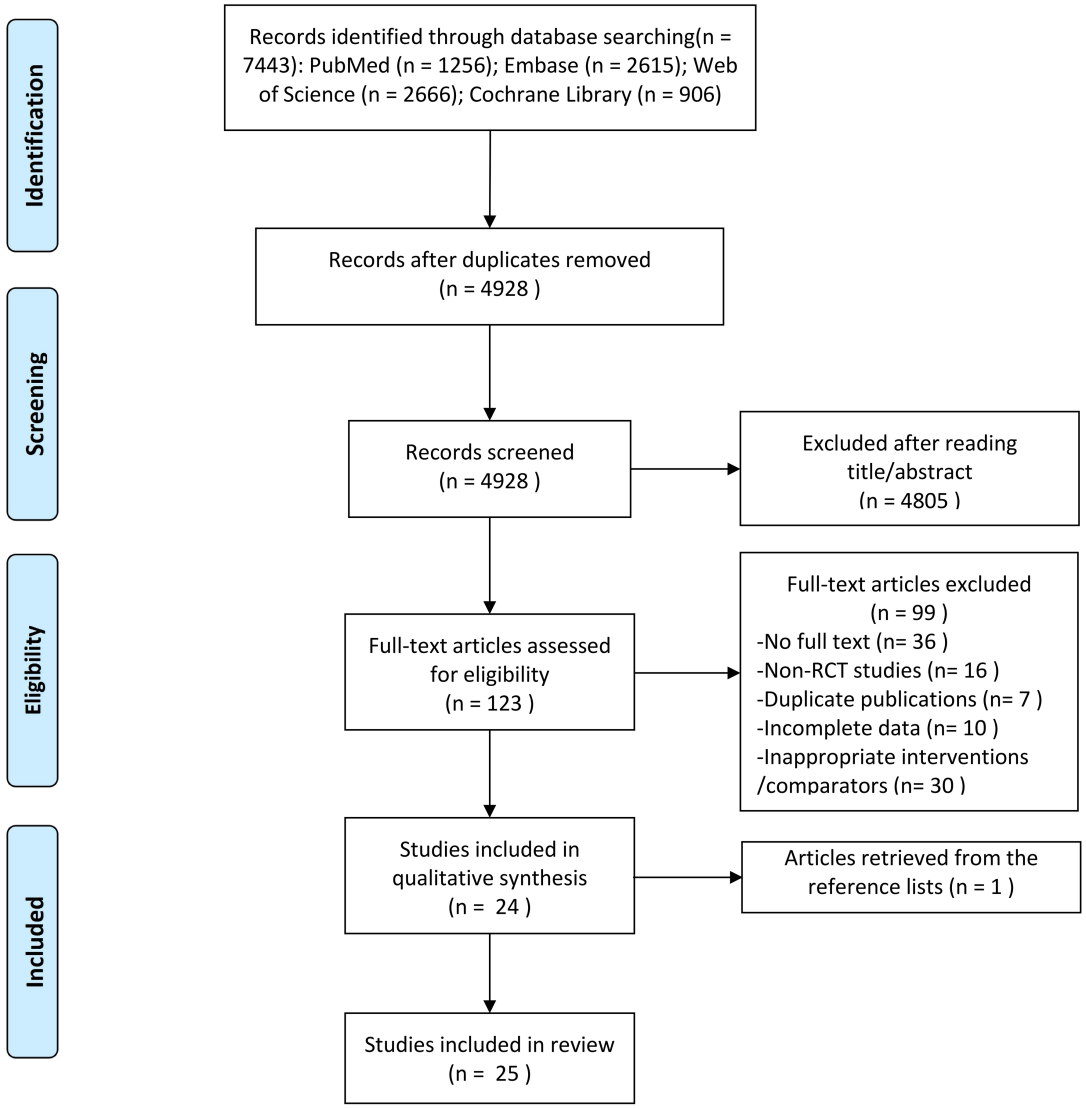
PRISMA flow diagram of the study selection.

According to the predefined search strategy, a total of 7,443 records were initially identified. After removal of duplicates, the titles and abstracts of the remaining records were screened, and 123 studies were selected for full-text assessment. Of these, 24 studies met the inclusion and exclusion criteria. In addition, one additional study was identified through manual screening of reference lists from relevant original articles and review papers. Ultimately, a total of 25 studies were included in the network meta-analysis. The study selection process and inclusion details are presented in Figure 1.

### 3.2 Characteristics of included studies

A total of 25 RCTs were included, involving 991 participants, with 514 allocated to the intervention group and 477 to the control group. According to intervention type, 3 trials evaluated WLD, 11 evaluated ET, 4 evaluated RMT, and 6 evaluated OMT. One trial used a three-arm randomized design comparing RMT, OMT, and a control group. Detailed bibliographic characteristics of all included studies are presented in Table 1.

**Table 1:** The basic characteristics of included studies.

Study	Country	Sample size (EG/CG)	Age (years, EG/CG)	BMI (EG/CG)	AHI (EG/CG)	Interventions (EG/CG)	Frequency and duration	Outcomes
Kemppainen et al. (46)	Finland	52 (26/26)	51.0 ± 8.3/49.0 ± 8.9	33 ± 3.3/32 ± 3.1	11 ± 3.6/9 ± 2.7	WLD/CG	3 months	①
Tuomilehto et al. (47)	Finland	81 (40/41)	51.8 ± 9.0/50.9 ± 8.6	33.4 ± 2.8/31.4 ± 2.7	10.0 ± 3.0/9.3 ± 3.0	WLD/CG	12 weeks	①②④
Fernandes et al. (48)	Brazil	29 (14/15)	39.09 ± 3.26/44.10 ± 1.95	34.60 ± 0.80/35.92 ± 0.91	26.67 ± 9.41/16.88 ± 2.77	WLD/CG	16 weeks	①④
Kline et al. (27)	America	43 (27/16)	47.6 ± 1.3/45.9 ± 2.2	35.5 ± 1.2/33.6 ± 1.4	32.2 ± 5.6/24.4 ± 5.6	ET/CG	2 times/week, 12 weeks	①③
Desplan et al. (28)	France	26 (13/13)	NA	29.9 ± 3.4/31.3 ± 2.5	40.6 ± 19.4/39.8 ± 19.2	ET/CG	120 min/time, 6 times/week, 4 weeks	①②③④
Berger et al. (29)	France	96 (48/48)	NA	28.4 ± 4.3/28.5 ± 4.5	21.9 ± 7.0/21.0 ± 6.3	ET/CG	60 min/time, 3 times/week, 9 months	①④
Yang et al. (23)	China	70 (35/35)	46.3 ± 6.4/48.6 ± 7.2	27.6 ± 4.7/27.1 ± 3.5	20.2 ± 7.5/19.5 ± 6.1	ET/CG	30 min/time, 3 times/week, 12 weeks	①④
Yilmaz Gokmen et al. (24)	Turkey	50 (25/25)	50.44 ± 8.38/45.68 ± 7.64	30.56 ± 2.99/29.21 ± 3.49	19.32 ± 7.09/18.66 ± 6.14	ET/CG	60 min/time, 3 times/week, 12 weeks	①②③④
Guerra et al. (30)	Brazil	44 (22/22)	53 ± 2/50 ± 1	29.6 ± 0.9/29.5 ± 0.8	44 ± 7/44 ± 6	ET/CG	30–40 min/time, 3 times/week, 6 months	①④
Jurado-García et al. (25)	Spain	68 (34/34)	52 ± 6.6/50 ± 9.5	32 ± 5.2/32 ± 4.3	29 ± 19.7/27 ± 10.4	ET/CG	6 months	①②④
Bughin et al. (31)	France	68 (34/34)	53.71(9.86)/55.00(10.27)	30.65(6.20)/30.60(3.40)	28.15(12.89)/26.10(15.78)	ET/CG	60 min/time, 3 times/week, 8 weeks	①②
Goya et al. (32)	Brazil	44 (22/22)	53.8 ± 1.7/49.3 ± 1.7	29.3 ± 0.9/29.7 ± 0.9	45.8 ± 7.8/39.8 ± 5.5	ET/CG	40–50 min/time, 3 times/week, 40 weeks	①④
Ueno-Pardi et al. (33)	Brazil	50 (25/25)	53 ± 7/51 ± 6	30.2 ± 3.8/29.3 ± 3.1	45 ± 29/41 ± 24	ET/CG	60 min/time, 3 times/week, 6 months	①④
Lins-Filho et al. (26)	Brazil	42 (21/21)	53.2 ± 9.9/55.1 ± 9.8	34.5 ± 6.6/33.8 ± 5.0	35.6 ± 3.9/49.3 ± 6.2	ET/CG	35 min/time, 3 times/week, 12 weeks	①③④
Vranish and Bailey (49)	America	26 (13/13)	61.5 ± 3.9/69.1 ± 3.4	27.0 ± 1.0/28.5 ± 1.6	21.9 ± 4.4/29.9 ± 8.9	RMT/CG	5 min/time, 1 time/day, 6 weeks	①④
Souza et al. (50)	Brazil	30 (15/15)	54.8 ± 6.9/49.9 ± 11.6	NA	27.6 ± 11.9/34.0 ± 18.4	RMT/CG	15 min/time, 2 times/day, 12 weeks	②③
Nóbrega-Júnior et al. (51)	Brazil	35 (18/17)	58.6 ± 5.6/60.1 ± 2.7	33.4 (4.2)/32.7 (11.1)	31.7 ± 15.9/31.4 ± 20.8	RMT/CG	2 times/day, 8 weeks	①②③
Azeredo et al. (52)	Brazil	43 (22/21)	63 ± 15/55 ± 15	29.8 ± 5.2/31.2 ± 5.2	29 (13)/20 (14)	RMT/CG	1 time/day, 12 weeks	①②③④
Puhan et al. (53)	Switzerland	25 (14/11)	49.9 ± 6.7/47.0 ± 8.9	25.8 ± 4.0/25.9 ± 2.4	22.3 ± 5.0/19.9 ± 4.7	OMT/CG	25 min/time, 4 months	①②③
Guimarães et al. (54)	Brazil	31 (16/15)	51.5 ± 6.8/47.7 ± 9.8	29.6 ± 3.8/31.0 ± 2.8	22.4 ± 4.8/22.4 ± 5.4	OMT/CG	30 min/time, 1 time/day, 3 months	①②③④
Diaféria et al. (55)	Brazil	51 (27/24)	45.2 ± 13.0/42.9 ± 10.5	25.0 ± 7.4/28.6 ± 4.0	28.0 ± 22.7/27.8 ± 20.3	OMT/CG	20 min/time, 3 times/day, 3 months	①②④
Kim et al. (44)	Korea	31 (16/15)	53.88 ± 18.44/49.20 ± 19.40	24.44 ± 2.88/26.78 ± 4.88	19.51 ± 11.41/16.57 ± 7.28	OMT/CG	12 weeks	①②③
O'Connor-Reina et al. (56)	Spain	40 (20/20)	45.9 (35.6)/50.26 (28.0)	28.9 (4.22)/29.6 (4.98)	44.77 (21.85)/47.36 (17.54)	OMT/CG	20 min/time, 1 time/day, 3 months	①②③④
Poncin et al. (57)	Switzerland	27 (14/13)	48.0 (10.7)/56.0 (11.0)	26.5 (5.1)/28.9 (12.0)	18.9 (27.2)/16.8 (22.0)	OMT/CG	15 min/time, 4 times/week, 6 weeks	①②③④
Erturk et al. (58)	Turkey	54 (18/18/18)	49.66 ± 9.08/53.71 ± 7.08/47.25 ± 7.32	31.00 ± 5.42/31.36 ± 3.84/32.06 ± 3.69	30.08 ± 19.33/42.60 ± 27.10/38.70 ± 23.98	RMT/OMT/CG	12 weeks	①②③

EG, experimental group, NA, not available, ① AHI, ② ESS, ③ PSQI, ④ BMI; Age, BMI, and AHI were expressed as mean ± standard deviation (SD) or median (interquartile range, IQR).

### 3.3 Risk of bias assessment and evidence quality evaluation

**Figure 2:**
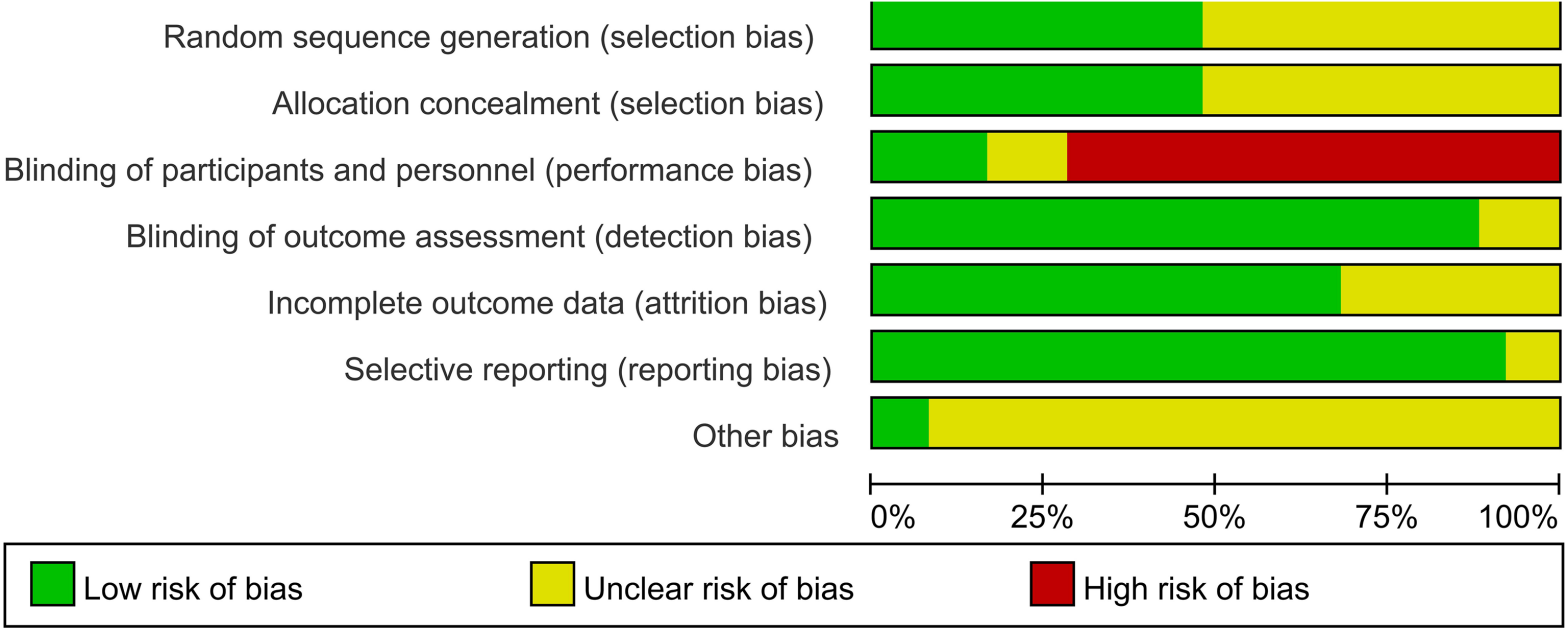
Risk of bias graph.

The results of the risk of bias assessment are summarized in Figure 2. Among the 25 included RCTs, 12 studies reported appropriate methods for random sequence generation, whereas reporting of allocation concealment was insufficient, with only 12 studies describing specific concealment procedures. Owing to the nature of the interventions, blinding of participants and study personnel was difficult to implement and was therefore judged as high risk in most studies. In contrast, blinding of outcome assessors was adequately addressed in 22 studies. Regarding completeness of outcome data, most studies reported complete outcome data. The overall risk of selective reporting was low, with only a few studies providing insufficient information. Other sources of bias were mostly judged as unclear. Detailed risk of bias assessments for each study are provided in Appendix 3.

Due to limitations in blinding participants and outcome assessors in the included randomized controlled trials, we downgraded the overall quality of the evidence by one level. This grading system categorizes evidence quality from very low to moderate, with specific ratings for each outcome detailed in Appendix 4.

### 3.4 Assessment of inconsistency

For outcomes with closed loops in the network (AHI, ESS, and PSQI), results from inconsistency testing, node-splitting analyses, and loop inconsistency tests indicated no statistically significant differences between direct and indirect evidence (all *p* > 0.05), suggesting no evidence of inconsistency. For BMI, the network structure did not form closed loops; therefore, inconsistency could not be assessed and a consistency model was applied. Overall, within the assessable range, no significant inconsistency was observed in the network meta-analysis. Detailed results are provided in Appendix 5.

### 3.5 Network meta-analysis

**Figure 3:**
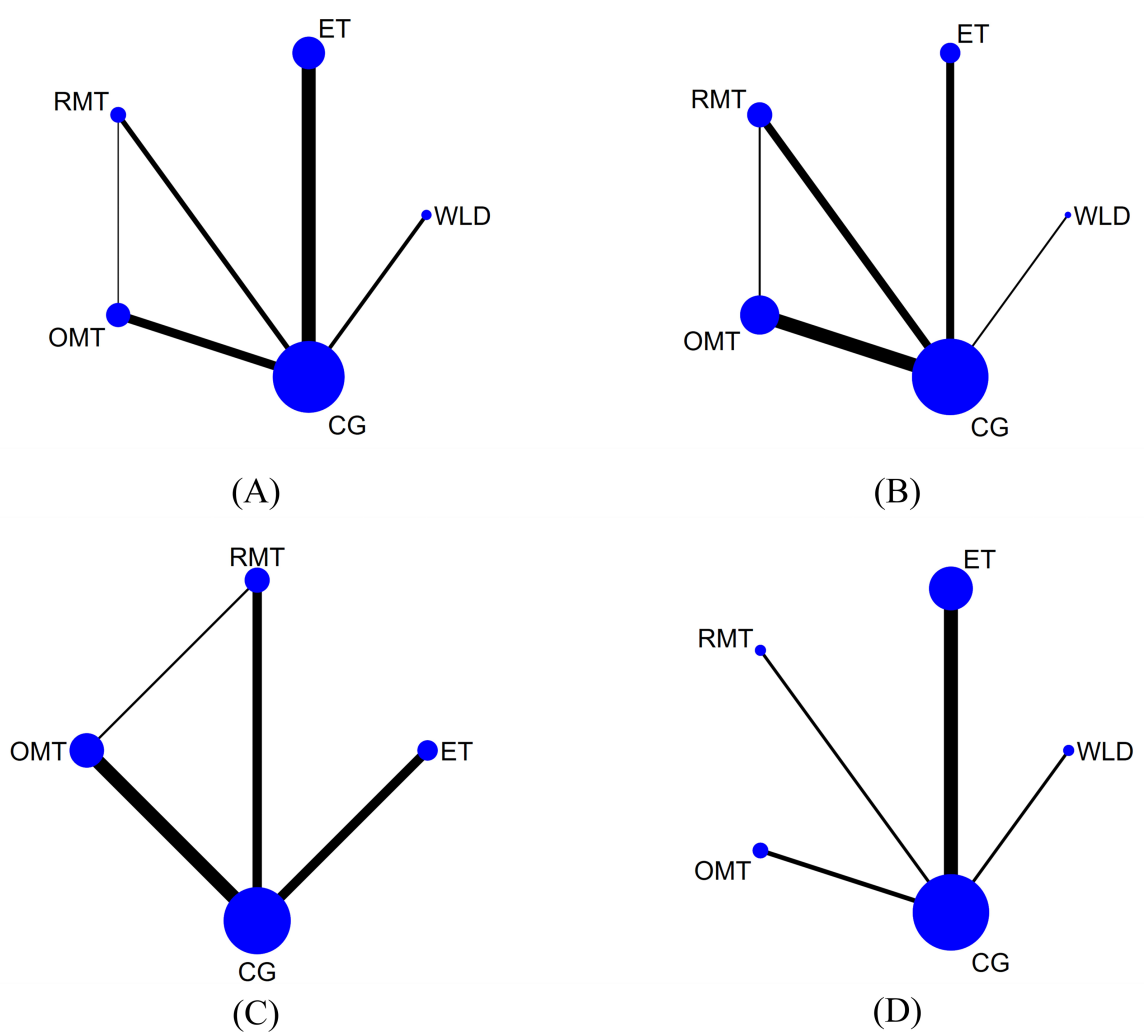
Network evidence plot. **(A)** AHI, **(B)** ESS, **(C)** PSQI, **(D)** BMI.

**Figure 4:**
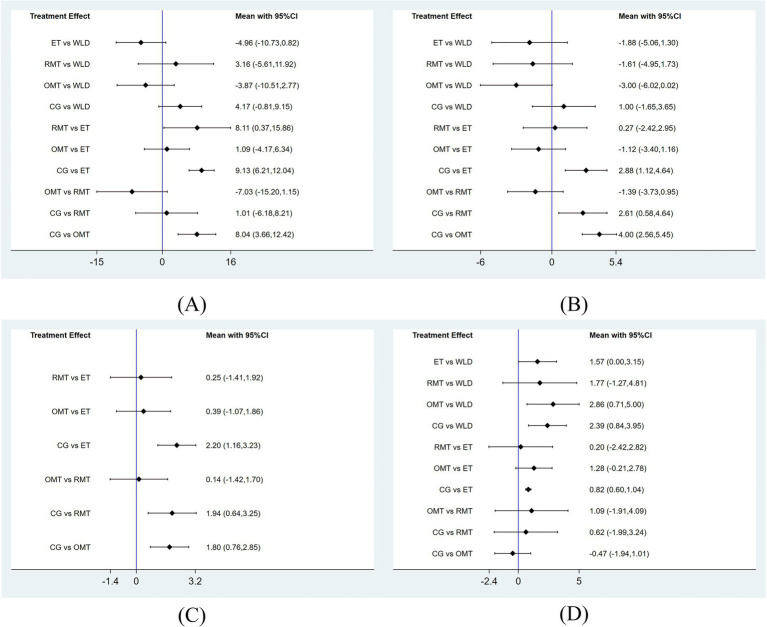
Forest plots. **(A)** AHI, **(B)** ESS, **(C)** PSQI, **(D)** BMI.

For AHI, a total of 24 studies involving 975 participants were included in the analysis. The network relationships between these interventions are shown in Figure 3A. Initially, each intervention was compared with the CG through conventional meta-analysis. The results showed that WLD (MD = −3.87, 95% CI: −6.22 to −1.52; *I*^2^ = 0.0%), ET (MD = −9.09, 95% CI: −12.56 to −5.63; *I*^2^ = 81.0%), and OMT (MD = −8.33, 95% CI: −11.35 to −5.30; *I*^2^ = 42.0%) showed statistically significant improvements, as detailed in Appendix 6. The results of the network meta-analysis indicated that, compared with the control group, ET (MD = −9.13, 95% CI: −12.04 to −6.21) and OMT (MD = −8.04, 95% CI: −12.42 to −3.66) significantly reduced AHI in patients with OSA. In addition, ET showed significantly greater efficacy than RMT in reducing AHI (MD = 8.11, 95% CI: 0.37 to 15.86) (Figure 4A, Appendix 7). According to the SUCRA rankings, the interventions were ordered as follows for reduction in AHI: ET (89.9%), OMT (79.4%), WLD (46.9%), RMT (22.9%), and CG (10.8%). Detailed results are presented in Table 2, Appendix 8.

**Table 2:** Ranking table of SUCRA values.

Intervention	AHI	ESS	PSQI	BMI
WLD	46.9%	27.2%	NA	96.2%
ET	89.9%	65.4%	78.1%	63.5%
RMT	22.9%	59.4%	64.3%	49.9%
OMT	79.4%	92.2%	57.6%	13.8%
CG	10.8%	5.8%	0.1%	26.6%

NA, not available.

For ESS, 15 studies comprising 558 participants were included in the analysis. The network relationships between these interventions are shown in Figure 3B. Initially, each intervention was compared with the CG through conventional meta-analysis. The results showed that ET (MD = −2.60, 95% CI: −3.84 to −1.36; *I*^2^ = 46.0%), RMT (MD = −2.58, 95% CI: −4.37 to −0.79; *I*^2^ = 38.7%), and OMT (MD = −4.18, 95% CI: −5.35 to −3.00; *I*^2^ = 17.3%) showed statistically significant improvements, as detailed in Appendix 6. The results of the network meta-analysis indicated that, compared with the control group, ET (MD = −2.88, 95% CI: −4.64 to −1.12), RMT (MD = −2.61, 95% CI: −4.64 to −0.58), and OMT (MD = −4.00, 95% CI: −5.45 to −2.56) were all associated with significant improvements in daytime sleepiness among patients with OSA (Figure 4B, Appendix 7). According to the SUCRA rankings, the interventions were ordered as follows for reduction in ESS: OMT (92.2%), ET (65.4%), RMT (59.4%), WLD (27.2%), and CG (5.8%). Detailed results are presented in Table 2, Appendix 8.

For PSQI, a total of 13 studies involving 397 participants were included in the analysis. The network relationships between these interventions are shown in Figure 3C. Initially, each intervention was compared with the CG through conventional meta-analysis. The results showed that ET (MD = −2.20, 95% CI: −3.24 to −1.16; *I*^2^ = 0.0%), RMT (MD = −2.03, 95% CI: −3.39 to −0.67; *I*^2^ = 0.0%), and OMT (MD = −1.88, 95% CI: −2.95 to −0.82; *I*^2^ = 23.4%) all showed statistically significant improvements, as detailed in Appendix 6. The results of the network meta-analysis indicated that, compared with the control group, ET (MD = −2.20, 95% CI: −3.23 to −1.16), RMT (MD = −1.94, 95% CI: −3.25 to −0.64), and OMT (MD = −1.80, 95% CI: −2.85 to −0.76) were all associated with significant improvements in sleep quality among patients with OSA (Figure 4C, Appendix 7). According to the SUCRA rankings, the interventions were ordered as follows for reduction in PSQI: ET (78.1%), RMT (64.3%), OMT (57.6%), and CG (0.1%). Detailed results are presented in Table 2, Appendix 8.

For BMI, 17 studies involving 718 participants were included in the analysis. The network relationships between these interventions are shown in Figure 3D. Initially, each intervention was compared with the CG through conventional meta-analysis. The results showed that WLD (MD = −2.43, 95% CI: −3.99 to −0.88; *I*^2^ = 0.0%) and ET (MD = −0.81, 95% CI: −0.94 to −0.69; *I*^2^ = 22.7%) both showed statistically significant improvements, as detailed in Appendix 6. The results of the network meta-analysis indicated that, compared with the control group, WLD (MD = −2.39, 95% CI: −3.95 to −0.84) and ET (MD = −0.82, 95% CI: −1.04 to −0.60) were associated with significant reductions in BMI. In addition, WLD demonstrated significantly greater effects on BMI reduction than OMT (MD = −2.86, 95% CI: −5.00 to −0.71) (Figure 4D, Appendix 7). According to the SUCRA rankings, the interventions were ordered as follows for reduction in BMI: WLD (96.2%), ET (63.5%), RMT (49.9%), CG (26.6%), and OMT (13.8%). Detailed results are presented in Table 2, Appendix 8.

### 3.6 Subgroup analysis

We performed subgroup analyses of all ET studies, which were categorized by exercise modality into the aerobic exercise alone (AE) group (23–26) and the combined AE and resistance training (RT) group (27–33). Based on the effect sizes of each intervention relative to the control group and the SUCRA ranking results, combined AE and RT was the most effective in reducing AHI, whereas AE alone was ranked highest for improving PSQI. The relative rankings of the other interventions are detailed in Appendix 9.

### 3.7 Sensitivity analysis

To assess the robustness of the main results, we conducted a sensitivity analysis to evaluate the potential impact of small-sample studies on the pooled effect size. Based on predefined criteria, we excluded all RCTs with a sample size of less than 10 per group, reconstructed the network, and performed a network meta-analysis on the four primary outcomes: AHI, ESS, PSQI, and BMI. The analysis results showed that after excluding small-sample studies, the direction of effect size and statistical significance for each intervention relative to the control group did not change substantially. Furthermore, the intervention efficacy rankings based on SUCRA values were also consistent with the main analysis results, as detailed in Appendix 10.

### 3.8 Publication bias analysis

**Figure 5:**
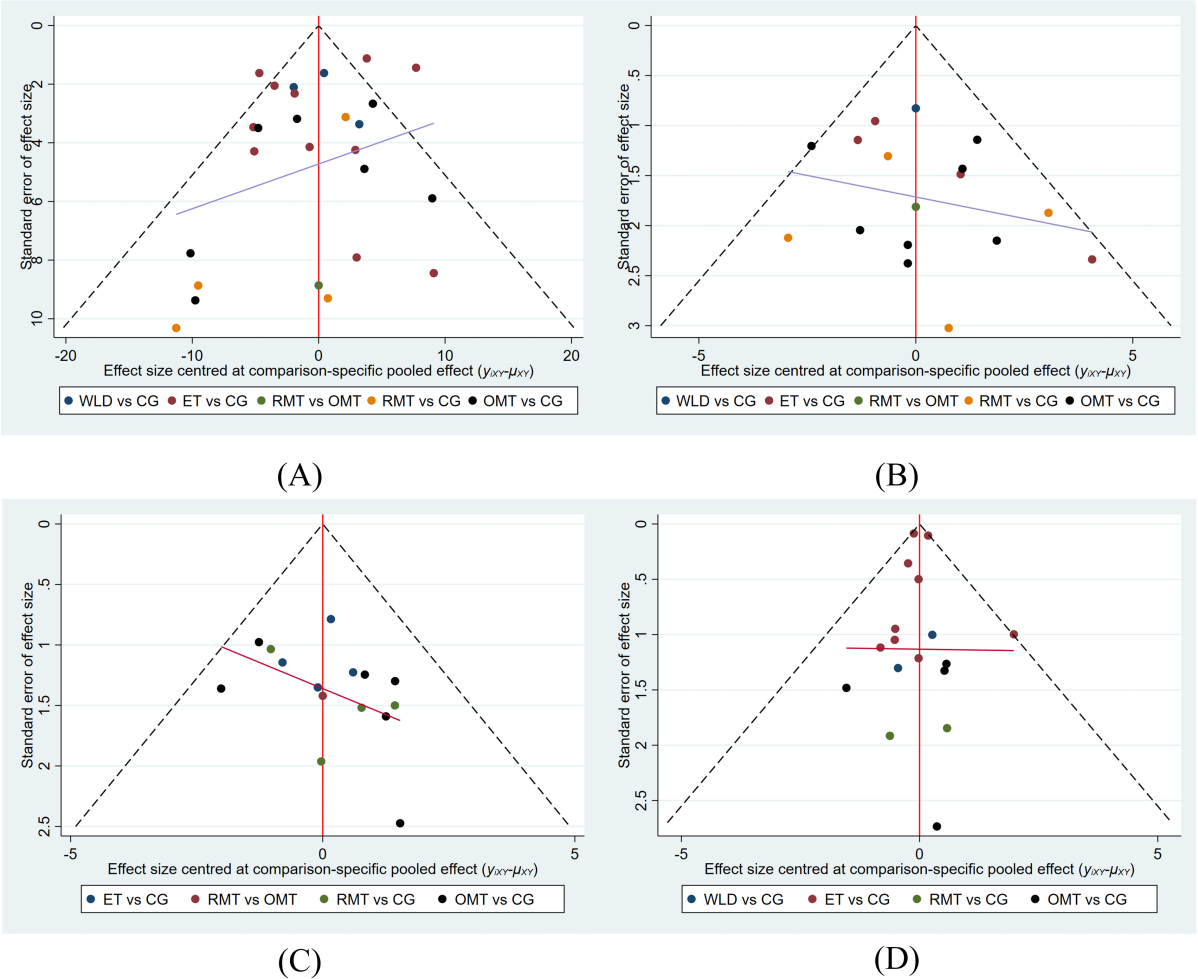
Funnel plots. **(A)** AHI, **(B)** ESS, **(C)** PSQI, **(D)** BMI.

Publication bias was assessed for the four outcomes of AHI, ESS, PSQI, and BMI, and the corresponding funnel plots are presented in Figure 5. Overall, the scatter points in the funnel plots were approximately symmetrically distributed on both sides of the inverted funnel and were largely centered around the midline, showing a relatively balanced dispersion pattern. No obvious asymmetry was observed, suggesting a low likelihood of small-study effects or publication bias in the present study. Egger’s test did not find strong evidence of publication bias for any outcome (AHI: *p* = 0.066; ESS: *p* = 0.120; PSQI: *p* = 0.375; BMI: *p* = 0.834). Detailed results are provided in Appendix 11.

## 4 Discussion

In this network meta-analysis, the relative effects of WLD, ET, OMT, and RMT were evaluated in patients with OSA. A total of 25 RCTs involving 991 participants were included. The findings indicate that these interventions exert differential effects across clinical outcomes. ET demonstrated the greatest efficacy in reducing the AHI and PSQI scores; OMT showed the most pronounced improvement in ESS scores, while WLD was most effective in lowering BMI scores. These findings suggest that lifestyle and functional training interventions may act through distinct pathophysiological pathways in OSA.

The AHI level in patients with OSA reflects the degree of upper airway obstruction and the severity of apneic events (34). This study shows that ET can significantly reduce AHI, ESS, PSQI, and BMI in patients with OSA. Notably, although WLD demonstrated the greatest effect on reducing BMI, exercise training showed superior efficacy in improving AHI, suggesting that improvements in AHI may not be entirely dependent on weight loss, a notion that is also supported by prior evidence (35). It has been proposed that exercise may enhance lower limb venous return and reduce fluid retention, limiting the nocturnal rostral fluid shift toward the neck and peripharyngeal tissues in the supine position, which in turn alleviates airway compression and narrowing, improving upper airway patency (36). Additionally, OSA is commonly associated with systemic low-grade inflammation and oxidative stress induced by chronic intermittent hypoxia. Regular exercise has well-established anti-inflammatory and antioxidant effects, which may downregulate circulating inflammatory mediators, reduce inflammatory edema of the upper airway mucosa, and improve local neuromuscular function (37, 38). Furthermore, subgroup analysis in this study showed that, in terms of improving AHI, AE combined with RT was most effective, while AE alone showed the best efficacy in improving PSQI. Current research suggests that this may be related to the better effect of resistance training in enhancing leg muscle strength, as leg muscles play a core role in venous return and help reduce fluid retention in the legs (39). However, resistance training may, to some extent, increase body fatigue and muscle soreness, which could negatively affect subjective sleep comfort, thus slightly reducing its effect on PSQI compared to aerobic exercise alone.

The ESS is used to assess daytime sleepiness and sleep-related functional impairment in patients with OSA and represents a patient-reported, symptom-based measure of subjective experience (40). OMT may directly enhance the strength and coordination of the tongue, soft palate, and pharyngeal muscles, thereby enhancing the stability of upper airway dilator muscles during sleep. This stabilization can reduce the propensity for upper airway collapse and decrease arousal burden, leading to improved sleep restorative quality and, consequently, reduced daytime sleepiness as reflected by lower ESS scores (41, 42). In addition, previous studies have shown that a 3-month course of OMT not only improves upper airway function but is also associated with a significant reduction in neck circumference, further alleviating daytime sleepiness and snoring severity (43). As a high-frequency behavioral intervention that requires long-term adherence, good compliance may also enhance patients’ perception of symptom improvement, which may be reflected as more pronounced effects on subjective outcome measures such as ESS (44).

The specific rankings revealed by this network meta-analysis provide important insights for the personalized management of OSA, helping to prioritize intervention strategies based on the patients’ core symptoms and treatment goals. For patients whose primary goal is to reduce the frequency of respiratory events and improve sleep quality, ET should be the preferred conservative treatment, as it showed the most significant effect in reducing AHI and improving PSQI scores. However, considering the clinically meaningful thresholds for OSA patients (45) (where AHI ≥ 15 events/h is defined as the clinical cut-off, and a score of ≥2 points on the ESS and ≥3 points on the PSQI represent the Minimally Clinically Important Difference (MCID)), although ET (including AE and AE + RT) exerts a certain improving effect, its magnitude of improvement in AHI and PSQI fails to meet the corresponding clinical cut-off and MCID, resulting in limited clinical significance. If the patient’s main complaint is daytime excessive sleepiness, OMT may be the optimal choice. OMT directly improves airway collapse by enhancing the function and coordination of upper airway dilator muscles, and OMT, ET (including AE and AE combined with RT), and RMT all exceed the MCID for ESS, achieving clinical significance. For OSA patients with obesity, WLD should be the first-choice basic lifestyle intervention. Although conventional meta-analysis has shown that WLD can reduce BMI and significantly improve AHI, the network meta-analysis in this study did not observe significant improvements in AHI. This may be related to the limited number of relevant RCTs included, which may have affected the robustness and accuracy of evidence synthesis. Additionally, the MCID for BMI in OSA patients has not been clearly defined, and its clinical significance cannot be determined. However, these interventions still hold important clinical value, particularly for patients who are unsuitable for or intolerant of CPAP treatment, MAD therapy, or those who refuse surgery, as they can serve as core adjunctive therapies to improve symptoms.

Several limitations of the present study should be acknowledged. First, although all included studies were RCTs, the overall methodological quality was heterogeneous. Among the 25 trials, only 12 explicitly reported appropriate methods for random sequence generation, and information on allocation concealment was frequently insufficient. In addition, there was some heterogeneity in the control group. Due to the different nature of the interventions (e.g., ET is difficult to implement with strict blinding, while RMT often uses a sham training device as a placebo control), these differences may introduce heterogeneity in patient expectations and behavioral adherence, which could affect the effect estimates. Second, with respect to network structure, the BMI outcome did not form closed loops, precluding formal inconsistency testing; consequently, analyses were conducted using a consistency model only, limiting further assessment of agreement between direct and indirect evidence. In addition, the distribution of evidence across outcomes was imbalanced among interventions (e.g., WLD lacked PSQI data), which may have affected the robustness of effect estimates as well as the precision and stability of the ranking results. Finally, although SUCRA was used to provide relative rankings of intervention efficacy, these rankings may still be influenced by the number and quality of included studies and by the underlying network geometry, and should therefore be interpreted with caution. Future research will require more well-designed, adequately powered high-quality RCTs to further validate the specific advantages of different lifestyle and functional training interventions in various clinical outcomes of OSA.

## 5 Conclusion

ET was most effective in reducing AHI and PSQI scores, OMT yielded the greatest improvement in ESS scores, and WLD was most effective in lowering BMI. These findings indicate that lifestyle and functional training interventions exhibit distinct, outcome-specific effects in the management of OSA. Therefore, in clinical practice, individualized intervention strategies should be tailored based on patients’ predominant symptoms and specific therapeutic goals. However, the effects of these interventions still require further high-quality evidence to be fully validated.

## Data Availability

The original contributions presented in the study are included in the article/Supplementary material, further inquiries can be directed to the corresponding authors.
