# Supplementary material for: Comparative effects of weight-loss diet, exercise training, respiratory muscle training, and oropharyngeal muscle training in obstructive sleep apnea: a systematic review and network meta-analysis
Source: Front Med (Lausanne). 2026 Mar 19;13:1789371. doi: 10.3389/fmed.2026.1789371 (PMC13044032; doi:10.3389/fmed.2026.1789371)
Supplement: Supplementary file 1 [file Data_Sheet_1.docx]

**Supplementary Material**

**Appendix 1 Detailed searching strategies**

**1.1 Search strategy in PubMed（n = 1256）**

| #1 | ((((((((((((("Sleep Apnea, Obstructive"[Mesh]) OR (Apneas, Obstructive Sleep[Title/Abstract])) OR (Obstructive Sleep Apneas[Title/Abstract])) OR (Sleep Apneas, Obstructive[Title/Abstract])) OR (Apnea, Obstructive Sleep)) OR (Sleep Apnea Hypopnea Syndrome)) OR (Obstructive Sleep Apnea Syndrome)) OR (Obstructive Sleep Apnea)) OR (Syndrome, Obstructive Sleep Apnea)) OR (Syndrome, Sleep Apnea, Obstructive)) OR (Sleep Apnea Syndrome, Obstructive)) OR (OSAHS)) OR (Upper Airway Resistance Sleep Apnea Syndrome)) OR (Syndrome, Upper Airway Resistance, Sleep Apnea) |
| --- | --- |
| #2 | ((((((((((((((((((((((((("Exercise"[Mesh]) OR (Exercises[Title/Abstract])) OR (Exercise, Physical[Title/Abstract])) OR (Exercises, Physical[Title/Abstract])) OR (Physical Exercise[Title/Abstract])) OR (Physical Exercises[Title/Abstract])) OR (Exercise, Aerobic[Title/Abstract])) OR (Aerobic Exercise[Title/Abstract])) OR (Aerobic Exercises[Title/Abstract])) OR (Exercises, Aerobic[Title/Abstract])) OR (Exercise, Isometric[Title/Abstract])) OR (Exercises, Isometric[Title/Abstract])) OR (Isometric Exercises[Title/Abstract])) OR (Isometric Exercise[Title/Abstract])) OR (Acute Exercise[Title/Abstract])) OR (Acute Exercises[Title/Abstract])) OR (Exercise, Acute[Title/Abstract])) OR (Exercises, Acute[Title/Abstract])) OR (Exercise Training[Title/Abstract])) OR (Exercise Trainings[Title/Abstract])) OR (Training, Exercise[Title/Abstract])) OR (Trainings, Exercise[Title/Abstract])) OR (Physical Activity[Title/Abstract])) OR (Activities, Physical[Title/Abstract])) OR (Activity, Physical[Title/Abstract])) OR (Physical Activities[Title/Abstract]) |
| #3 | ("Diet"[Mesh]) OR (Diets[Title/Abstract]) |
| #4 | (((((("Breathing Exercises"[Mesh]) OR (Exercise, Breathing[Title/Abstract])) OR (Respiratory Muscle Training[Title/Abstract])) OR (Muscle Training, Respiratory[Title/Abstract])) OR (Training, Respiratory Muscle[Title/Abstract])) OR (Inspiratory Muscle Training[Title/Abstract])) OR (Expiratory Muscle Training[Title/Abstract]) |
| #5 | (((((((((((((((((((("Myofunctional Therapy"[Mesh]) OR (Myofunctional Therapies[Title/Abstract])) OR (Therapies, Myofunctional[Title/Abstract])) OR (Therapy, Myofunctional[Title/Abstract])) OR (Oral Myotherapy[Title/Abstract])) OR (Myotherapies, Oral[Title/Abstract])) OR (Myotherapy, Oral[Title/Abstract])) OR (Oral Myotherapies[Title/Abstract])) OR (Orofacial Myotherapy[Title/Abstract])) OR (Myotherapies, Orofacial[Title/Abstract])) OR (Myotherapy, Orofacial[Title/Abstract])) OR (Orofacial Myotherapies[Title/Abstract])) OR (Orofacial Myology[Title/Abstract])) OR (Myologies, Orofacial[Title/Abstract])) OR (Myology, Orofacial[Title/Abstract])) OR (Orofacial Myologies[Title/Abstract])) OR (Facial-Oral Tract Therapy[Title/Abstract])) OR (F.O.T.T.[Title/Abstract])) OR (oropharyngeal exercise[Title/Abstract])) OR (oropharyngeal exercises[Title/Abstract])) OR (oropharyngeal training[Title/Abstract]) |
| #6 | #2 OR #3 OR #4 OR #5 |
| #7 | #1 AND #6 |

**1.2 Search strategy in Embase（n = 2615）**

| #1 | 'sleep apnea, obstructive':ti,ab,kw OR 'apneas, obstructive sleep':ti,ab,kw OR 'obstructive sleep apneas':ti,ab,kw OR 'sleep apneas, obstructive':ti,ab,kw OR 'apnea, obstructive sleep':ti,ab,kw OR 'sleep apnea hypopnea syndrome':ti,ab,kw OR 'obstructive sleep apnea syndrome':ti,ab,kw OR 'obstructive sleep apnea':ti,ab,kw OR 'syndrome, obstructive sleep apnea':ti,ab,kw OR 'syndrome, sleep apnea, obstructive':ti,ab,kw OR 'sleep apnea syndrome, obstructive':ti,ab,kw OR osahs:ti,ab,kw OR 'upper airway resistance sleep apnea syndrome':ti,ab,kw OR 'syndrome, upper airway resistance, sleep apnea':ti,ab,kw |
| --- | --- |
| #2 | exercise:ti,ab,kw OR exercises:ti,ab,kw OR 'exercise, physical':ti,ab,kw OR 'exercises, physical':ti,ab,kw OR 'physical exercise':ti,ab,kw OR 'physical exercises':ti,ab,kw OR 'exercise, aerobic':ti,ab,kw OR 'aerobic exercise':ti,ab,kw OR 'aerobic exercises':ti,ab,kw OR 'exercises, aerobic':ti,ab,kw OR 'exercise, isometric':ti,ab,kw OR 'exercises, isometric':ti,ab,kw OR 'isometric exercises':ti,ab,kw OR 'isometric exercise':ti,ab,kw OR 'acute exercise':ti,ab,kw OR 'acute exercises':ti,ab,kw OR 'exercise, acute':ti,ab,kw OR 'exercises, acute':ti,ab,kw OR 'exercise training':ti,ab,kw OR 'exercise trainings':ti,ab,kw OR 'training, exercise':ti,ab,kw OR 'trainings, exercise':ti,ab,kw OR 'physical activity':ti,ab,kw OR 'activities, physical':ti,ab,kw OR 'activity, physical':ti,ab,kw OR 'physical activities':ti,ab,kw |
| #3 | diet:ti,ab,kw OR diets:ti,ab,kw |
| #4 | 'breathing exercises':ti,ab,kw OR 'exercise, breathing':ti,ab,kw OR 'respiratory muscle training':ti,ab,kw OR 'muscle training, respiratory':ti,ab,kw OR 'training, respiratory muscle':ti,ab,kw OR 'inspiratory muscle training':ti,ab,kw OR 'expiratory muscle training':ti,ab,kw |
| #5 | 'myofunctional therapy':ti,ab,kw OR 'myofunctional therapies':ti,ab,kw OR 'therapies, myofunctional':ti,ab,kw OR 'therapy, myofunctional':ti,ab,kw OR 'oral myotherapy':ti,ab,kw OR 'myotherapies, oral':ti,ab,kw OR 'myotherapy, oral':ti,ab,kw OR 'oral myotherapies':ti,ab,kw OR 'orofacial myotherapy':ti,ab,kw OR 'myotherapies, orofacial':ti,ab,kw OR 'myotherapy, orofacial':ti,ab,kw OR 'orofacial myotherapies':ti,ab,kw OR 'orofacial myology':ti,ab,kw OR 'myologies, orofacial':ti,ab,kw OR 'myology, orofacial':ti,ab,kw OR 'orofacial myologies':ti,ab,kw OR 'facial-oral tract therapy':ti,ab,kw OR f.o.t.t.:ti,ab,kw OR 'oropharyngeal exercise':ti,ab,kw OR 'oropharyngeal exercises':ti,ab,kw OR 'oropharyngeal training':ti,ab,kw |
| #6 | #2 OR #3 OR #4 OR #5 |
| #7 | #1 AND #6 |

**1.3 Search strategy in Web of science（n = 2666）**

| #1 | (((((((((((((TS=(Sleep Apnea, Obstructive)) OR TS=(Apneas, Obstructive Sleep)) OR TS=(Obstructive Sleep Apneas)) OR TS=(Sleep Apneas, Obstructive)) OR TS=(Apnea, Obstructive Sleep)) OR TS=(Sleep Apnea Hypopnea Syndrome)) OR TS=(Obstructive Sleep Apnea Syndrome)) OR TS=(Obstructive Sleep Apnea)) OR TS=(Syndrome, Obstructive Sleep Apnea)) OR TS=(Syndrome, Sleep Apnea, Obstructive)) OR TS=(Sleep Apnea Syndrome, Obstructive)) OR TS=(OSAHS)) OR TS=(Upper Airway Resistance Sleep Apnea Syndrome)) OR TS=(Syndrome, Upper Airway Resistance, Sleep Apnea) |
| --- | --- |
| #2 | (((((((((((((((((((((((((TS=(Exercise)) OR TS=(Exercises)) OR TS=(Exercise, Physical)) OR TS=(Exercises, Physical)) OR TS=(Physical Exercise)) OR TS=(Physical Exercises)) OR TS=(Exercise, Aerobic)) OR TS=(Aerobic Exercise)) OR TS=(Aerobic Exercises)) OR TS=(Exercises, Aerobic)) OR TS=(Exercise, Isometric)) OR TS=(Exercises, Isometric)) OR TS=(Isometric Exercises)) OR TS=(Isometric Exercise)) OR TS=(Acute Exercise)) OR TS=(Acute Exercises)) OR TS=(Exercise, Acute)) OR TS=(Exercises, Acute)) OR TS=(Exercise Training)) OR TS=(Exercise Trainings)) OR TS=(Training, Exercise)) OR TS=(Trainings, Exercise)) OR TS=(Physical Activity)) OR TS=(Activities, Physical)) OR TS=(Activity, Physical)) OR TS=(Physical Activities) |
| #3 | (TS=(Diet)) OR TS=(Diets) |
| #4 | ((((((TS=(Breathing Exercises)) OR TS=(Exercise, Breathing)) OR TS=(Respiratory Muscle Training)) OR TS=(Muscle Training, Respiratory)) OR TS=(Training, Respiratory Muscle)) OR TS=(Inspiratory Muscle Training)) OR TS=(Expiratory Muscle Training) |
| #5 | ((((((((((((((((((((TS=(Myofunctional Therapy)) OR TS=(Myofunctional Therapies)) OR TS=(Therapies, Myofunctional)) OR TS=(Therapy, Myofunctional)) OR TS=(Oral Myotherapy)) OR TS=(Myotherapies, Oral)) OR TS=(Myotherapy, Oral)) OR TS=(Oral Myotherapies)) OR TS=(Orofacial Myotherapy)) OR TS=(Myotherapies, Orofacial)) OR TS=(Myotherapy, Orofacial)) OR TS=(Orofacial Myotherapies)) OR TS=(Orofacial Myology)) OR TS=(Myologies, Orofacial)) OR TS=(Myology, Orofacial)) OR TS=(Orofacial Myologies)) OR TS=(Facial-Oral Tract Therapy)) OR TS=(F.O.T.T.)) OR TS=(oropharyngeal exercise)) OR TS=(oropharyngeal exercises)) OR TS=(oropharyngeal training) |
| #6 | #2 OR #3 OR #4 OR #5 |
| #7 | #1 AND #6 |

**1.4 Search strategy in Cochrane Library（n = 906）**

| #1 | (sleep apnea, obstructive):ti,ab,kw OR (apneas, obstructive sleep):ti,ab,kw OR (obstructive sleep apneas):ti,ab,kw OR (sleep apneas, obstructive):ti,ab,kw OR (apnea, obstructive sleep):ti,ab,kw |
| --- | --- |
| #2 | (sleep apnea hypopnea syndrome):ti,ab,kw OR (obstructive sleep apnea syndrome):ti,ab,kw OR (obstructive sleep apnea):ti,ab,kw OR (syndrome, obstructive sleep apnea):ti,ab,kw OR (syndrome, sleep apnea, obstructive):ti,ab,kw |
| #3 | (Sleep Apnea Syndrome, Obstructive):ti,ab,kw OR (OSAHS):ti,ab,kw OR (Upper Airway Resistance Sleep Apnea Syndrome):ti,ab,kw OR (Syndrome, Upper Airway Resistance, Sleep Apnea):ti,ab,kw |
| #4 | #1 OR #2 OR #3 |
| #5 | (Exercise):ti,ab,kw OR (Exercises):ti,ab,kw OR (Exercise, Physical):ti,ab,kw OR (Exercises, Physical):ti,ab,kw OR (Physical Exercise):ti,ab,kw |
| #6 | (Physical Exercises):ti,ab,kw OR (Exercise, Aerobic):ti,ab,kw OR (Aerobic Exercise):ti,ab,kw OR (Aerobic Exercises):ti,ab,kw OR (Exercises, Aerobic):ti,ab,kw |
| #7 | (Exercise, Isometric):ti,ab,kw OR (Exercises, Isometric):ti,ab,kw OR (Isometric Exercises):ti,ab,kw OR (Isometric Exercise):ti,ab,kw OR (Acute Exercise):ti,ab,kw |
| #8 | (Acute Exercises):ti,ab,kw OR (Exercise, Acute):ti,ab,kw OR (Exercises, Acute):ti,ab,kw OR (Exercise Training):ti,ab,kw OR (Exercise Trainings):ti,ab,kw |
| #9 | (Training, Exercise):ti,ab,kw OR (Trainings, Exercise):ti,ab,kw OR (Physical Activity):ti,ab,kw OR (Activities, Physical):ti,ab,kw OR (Activity, Physical):ti,ab,kw |
| #10 | (Physical Activities):ti,ab,kw |
| #11 | #5 OR #6 OR #7 OR #8 OR #9 OR #10 |
| #12 | (Diet):ti,ab,kw OR (Diets):ti,ab,kw |
| #13 | (Breathing Exercises):ti,ab,kw OR (Exercise, Breathing):ti,ab,kw OR (Respiratory Muscle Training):ti,ab,kw OR (Muscle Training, Respiratory):ti,ab,kw OR (Training, Respiratory Muscle):ti,ab,kw |
| #14 | (Inspiratory Muscle Training):ti,ab,kw OR (Expiratory Muscle Training):ti,ab,kw |
| #15 | #13 OR #14 |
| #16 | (Myofunctional Therapy):ti,ab,kw OR (Myofunctional Therapies):ti,ab,kw OR (Therapies, Myofunctional):ti,ab,kw OR (Therapy, Myofunctional):ti,ab,kw OR (Oral Myotherapy):ti,ab,kw |
| #17 | (Myotherapies, Oral):ti,ab,kw OR (Myotherapy, Oral):ti,ab,kw OR (Oral Myotherapies):ti,ab,kw OR (Orofacial Myotherapy):ti,ab,kw OR (Myotherapies, Orofacial):ti,ab,kw |
| #18 | (Myotherapy, Orofacial):ti,ab,kw OR (Orofacial Myotherapies):ti,ab,kw OR (Orofacial Myology):ti,ab,kw OR (Myologies, Orofacial):ti,ab,kw OR (Myology, Orofacial):ti,ab,kw |
| #19 | (Orofacial Myologies):ti,ab,kw OR (Facial-Oral Tract Therapy):ti,ab,kw OR (F.O.T.T.):ti,ab,kw OR (oropharyngeal exercise):ti,ab,kw OR (oropharyngeal exercises):ti,ab,kw |
| #20 | (oropharyngeal training):ti,ab,kw |
| #21 | #16 OR #17 OR #18 OR #19 OR #20 |
| #22 | #11 OR #12 OR #15 OR #21 |
| #23 | #4 AND #22 |

**Appendix 2 Data processing**

If a study provides only the baseline and endpoint means and standard deviations, the mean difference is calculated by subtracting the baseline mean from the endpoint mean.

| Baseline | Endpoint | Change |
| --- | --- | --- |
| Mean (B)  SD (B) | Mean (E)  SD (E) | Mean (C)  SD (C) |

Mean (C) = Mean (E) - Mean (B)

SD(C)= $\sqrt{\text{SD (B)}\text{2}\text{ }\text{+ SD}\text{ }\text{(}\text{E}\text{)}\text{2}\text{ }\text{− (2 × r}\text{ }\text{× SD}\text{ }\text{(B) × SD}\text{ }\text{(}\text{E}\text{))}}$ (r=0.5)

For studies that reported medians and interquartile ranges (IQRs), we approximated the median as the mean and estimated the standard deviation (SD) using the formula "IQR ÷ 1.35."

**Appendix 3 Result of the risk of bias assessment**

**
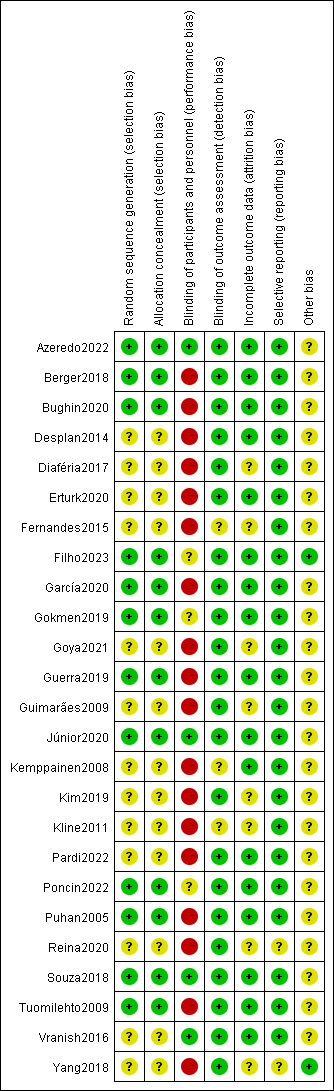
**

**Appendix 4 GRADE Assessment****.**

| Outcomes | Risk of Bias | Inconsistency | Indirectness | Imprecision | Other  Considerations | Quality |
| --- | --- | --- | --- | --- | --- | --- |
| Apnea-Hypopnea Index | No | No | No | No | Serious | Moderate |
| Epworth Sleepiness Scale | No | No | No | Serious | Serious | low |
| Pittsburgh Sleep Quality Index | No | No | No | Serious | Serious | low |
| Body Mass Index | No | No | Serious | Serious | Serious | Very low |

**Appendix 5 Assessment of inconsistency**

**5.1 Global inconsistency test**

|  | Chi²(2) | *P* |
| --- | --- | --- |
| AHI | 1.24 | 0.5378 |
| ESS | 2.84 | 0.2416 |
| PSQI | 1.34 | 0.5111 |

**5.2 Node-splitting method**

**5.2.1 Node-splitting method for AHI**

| **Side** | **Direct** | | **Indirect** | | **Difference** | | | **tau** |
| --- | --- | --- | --- | --- | --- | --- | --- | --- |
|  | **Coefficient** | **SE** | **Coefficient** | **SE** | **Coefficient** | **SE** | **P> \| z \|** |  |
| RMT-OMT | -6.38 | 9.62 | -7.20 | 4.68 | 0.82 | 10.73 | 0.94 | 3.79 |
| RMT-CG | 0.45 | 3.79 | 12.04 | 17.81 | -11.58 | 18.29 | 0.53 | 3.78 |
| OMT-CG | 7.90 | 2.26 | 17.26 | 18.28 | -9.36 | 18.42 | 0.61 | 3.76 |

**5.2.2 Node-splitting method for ESS**

| **Side** | **Direct** | | **Indirect** | | **Difference** | | | **tau** |
| --- | --- | --- | --- | --- | --- | --- | --- | --- |
|  | **Coefficient** | **SE** | **Coefficient** | **SE** | **Coefficient** | **SE** | **P> \| z \|** |  |
| RMT-OMT | 0.02 | 2.10 | -2.10 | 1.48 | 2.12 | 2.59 | 0.41 | 1.09 |
| RMT-CG | 2.63 | 1.12 | 2.41 | 4.36 | 0.22 | 4.58 | 0.96 | 1.18 |
| OMT-CG | 4.19 | 0.72 | -2.13 | 4.20 | 6.32 | 4.26 | 0.14 | 0.95 |

**5.2.3 Node-splitting method for PSQI**

| **Side** | **Direct** | | **Indirect** | | **Difference** | | | **tau** |
| --- | --- | --- | --- | --- | --- | --- | --- | --- |
|  | **Coefficient** | **SE** | **Coefficient** | **SE** | **Coefficient** | **SE** | **P> \| z \|** |  |
| RMT-OMT | 0.35 | 1.42 | 0.04 | 0.97 | 0.31 | 1.72 | 0.86 | 1.87e-06 |
| RMT-CG | 2.03 | 0.69 | 0.76 | 2.84 | 1.27 | 2.96 | 0.67 | 4.08e-08 |
| OMT-CG | 1.88 | 0.54 | -0.29 | 2.90 | 2.18 | 2.96 | 0.46 | 4.05e-08 |

**5.3 Loop inconsistency**

| Outcome | Loop | IF | SeIF | Z_value | P_value | CI_95 | Loop_Heterog_tau |
| --- | --- | --- | --- | --- | --- | --- | --- |
| AHI | RMT-OMT-CG | 1.903 | 10.324 | 0.184 | 0.854 | (0.00, 22.14) | 10.702 |
| ESS | RMT-OMT-CG | 1.015 | 2.509 | 0.405 | 0.686 | (0.00, 5.93) | 1.182 |
| PSQI | RMT-OMT-CG | 0.095 | 1.790 | 0.053 | 0.958 | (0.00, 3.60) | 0.239 |

CG = control group, WLD = weight-loss diet, ET = exercise training, RMT = respiratory muscle training, OMT = oropharyngeal muscle training, AHI = apnea–hypopnea index, ESS = Epworth Sleepiness Scale, PSQI = Pittsburgh Sleep Quality Index, BMI = body mass index. All abbreviations used in the table are consistent with the above definitions.

**Appendix 6 Meta-analysis**

**6.1 Forest Plot on AHI**

**6.1.1 Forest Plot of WLD on AHI**

**
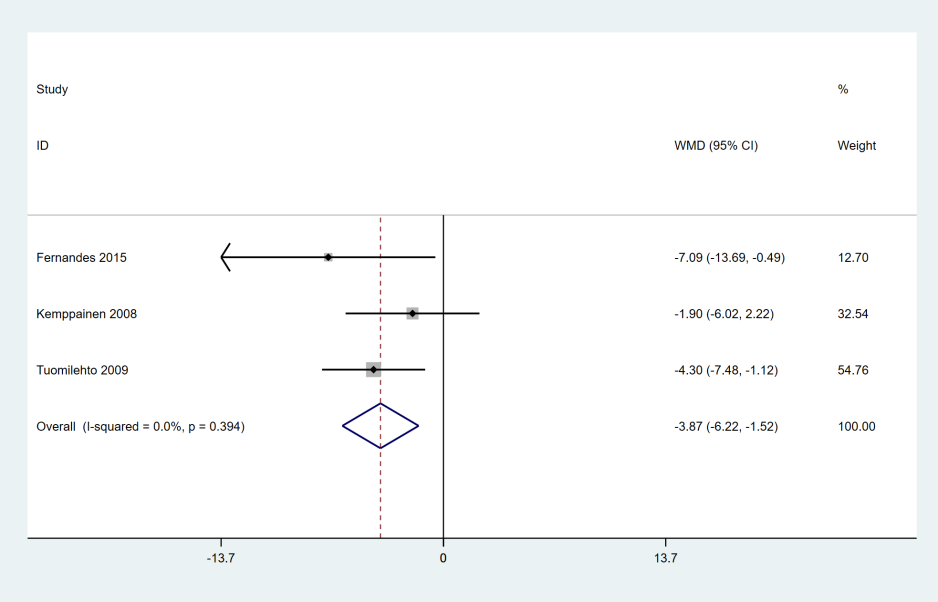
**

**6.1.2 Forest Plot of ET Effects on AHI**


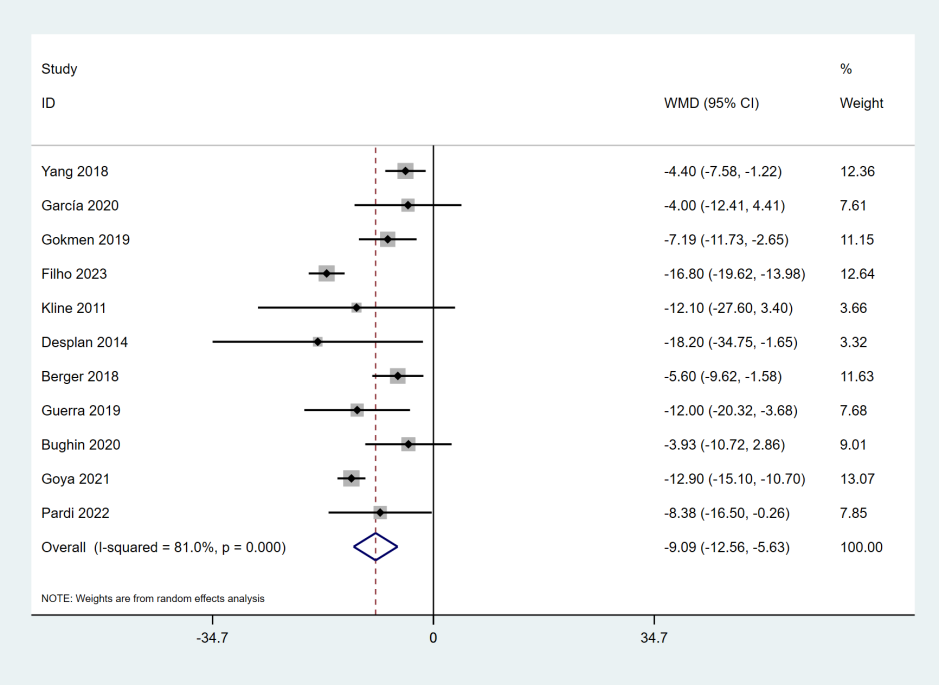


**6.1.3 Forest Plot of RMT on AHI**

**
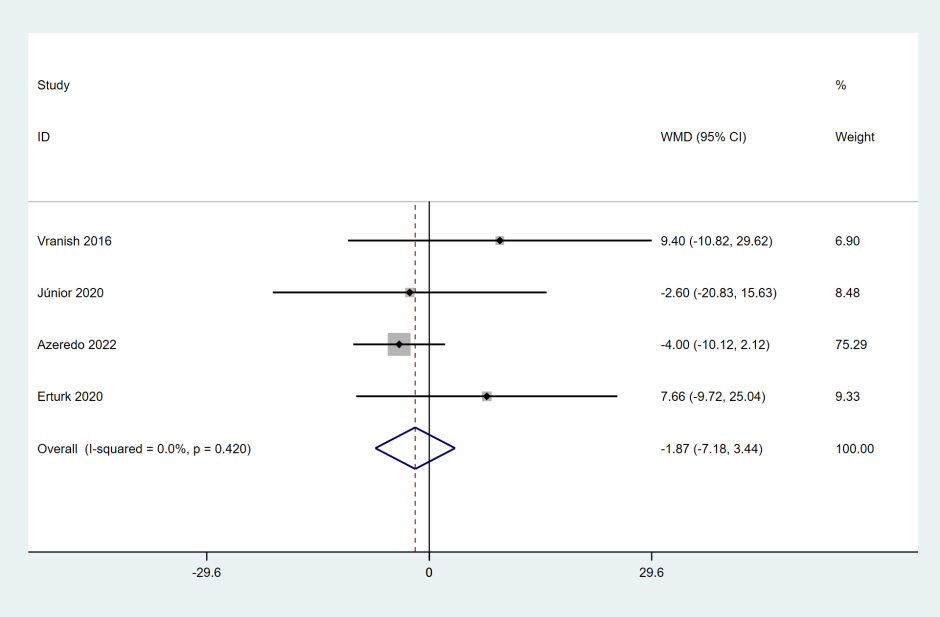
**

**6.1.4 Forest Plot of OMT on AHI**


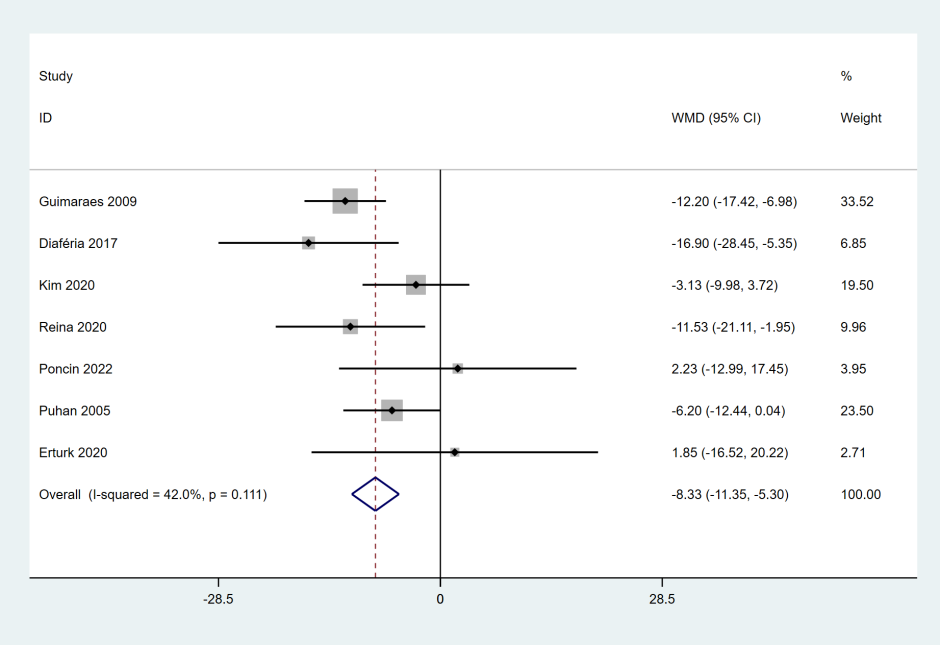


**6.2 Forest Plot on ESS**

**6.2.1 Forest Plot of ET Effects on ESS**


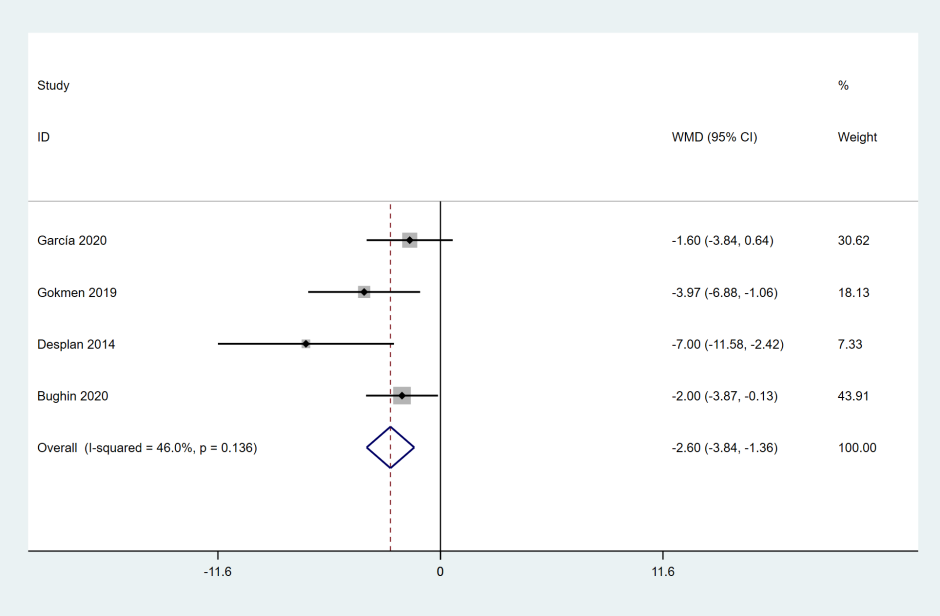


**6.2.2 Forest Plot of RMT on ESS**

**
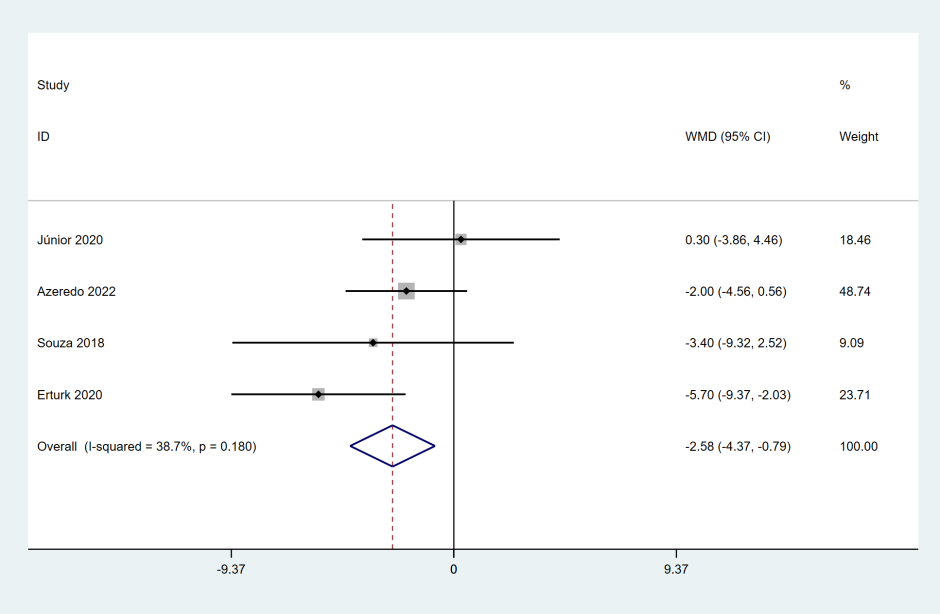
**

**6.2.3 Forest Plot of OMT on ESS**


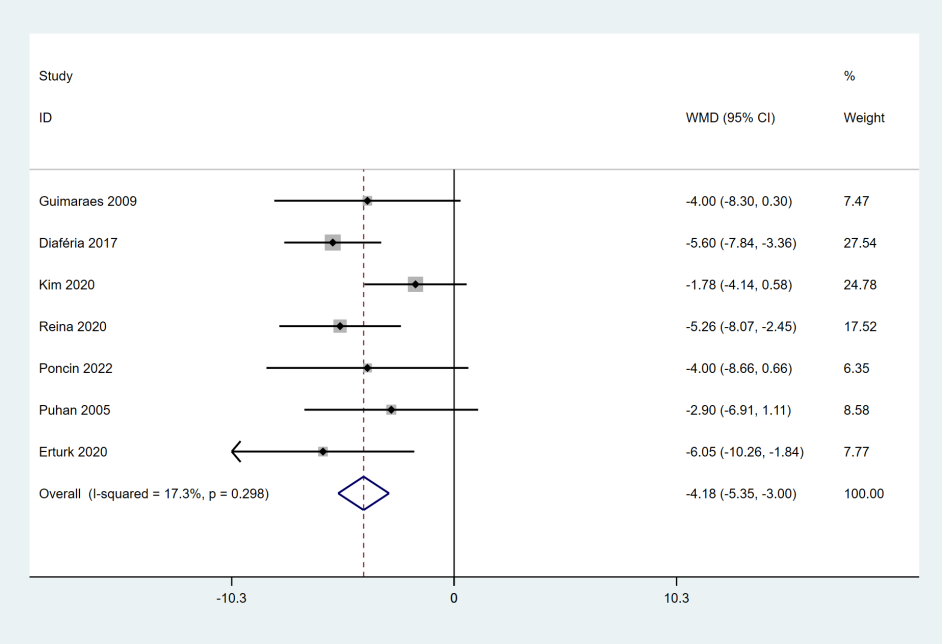


**6.3 Forest Plot on PSQI**

**6.3.1 Forest Plot of ET Effects on PSQI**


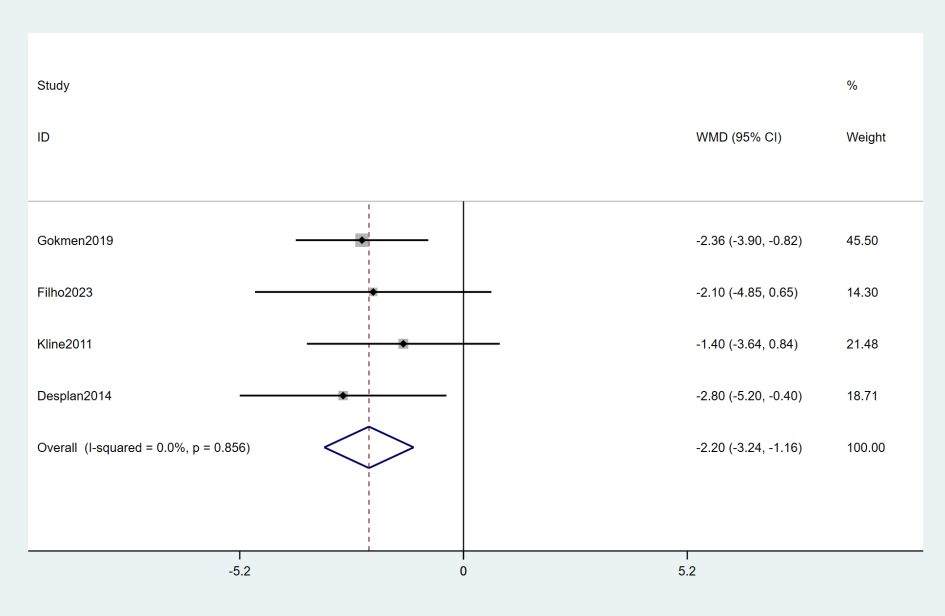


**6.3.2 Forest Plot of RMT on PSQI**

**
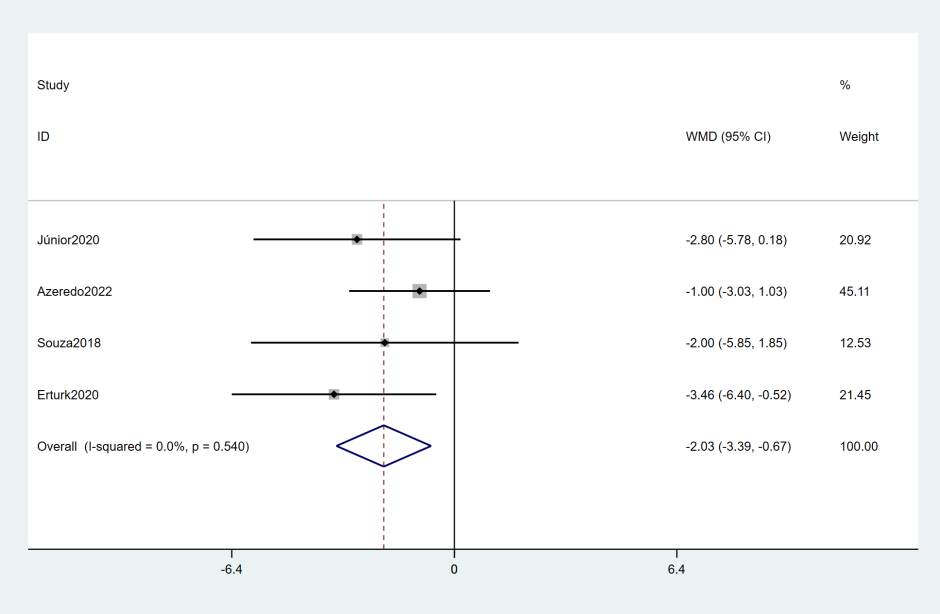
**

**6.3.3 Forest Plot of OMT on PSQI**


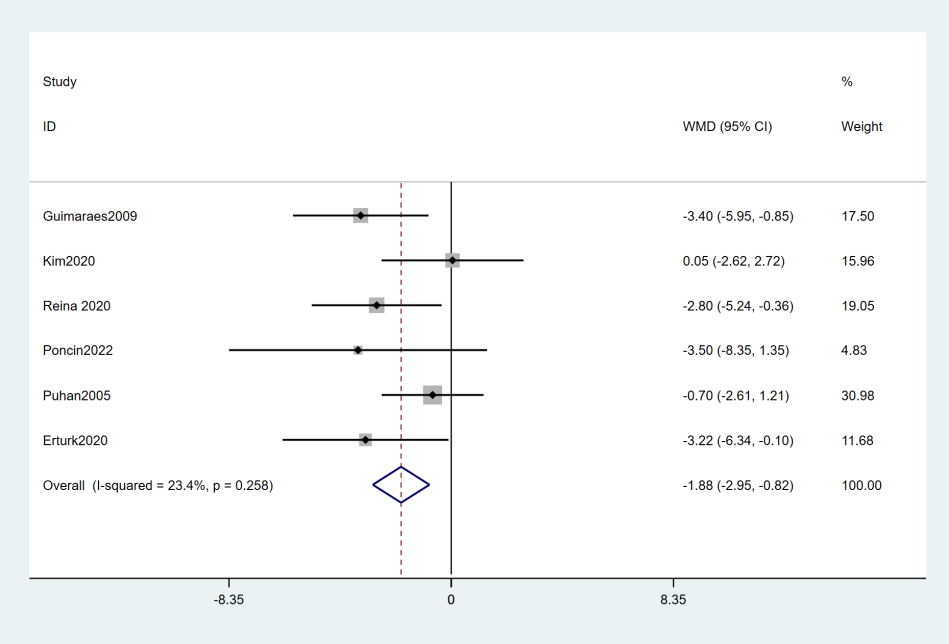


**6.4 Forest Plot on BMI**

**6.4.1 Forest Plot of WLD on BMI**

**
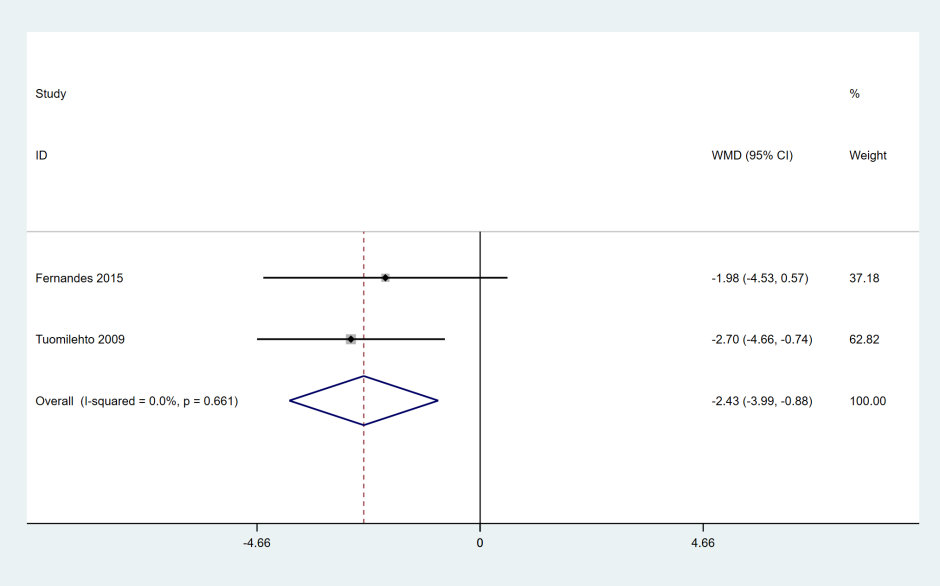
**

**6.4.2 Forest Plot of ET Effects on BMI**


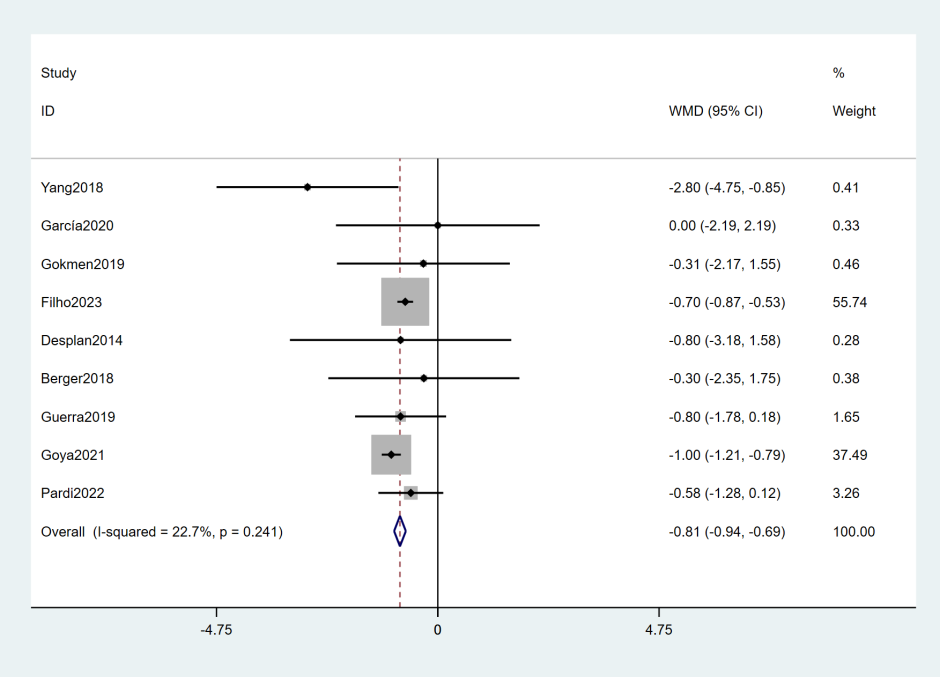


**6.4.3 Forest Plot of RMT on BMI**

**
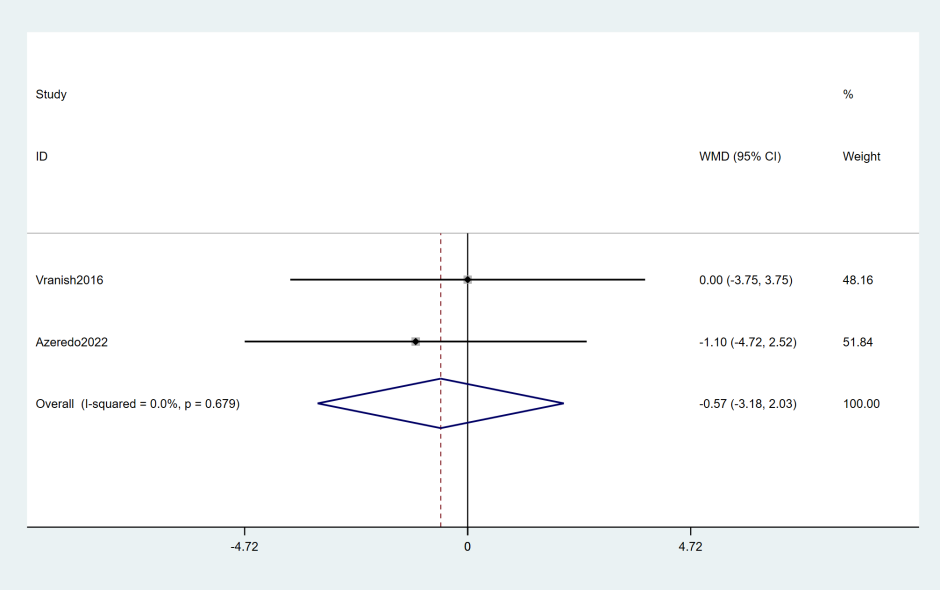
**

**6.4.4 Forest Plot of OMT on BMI**

**
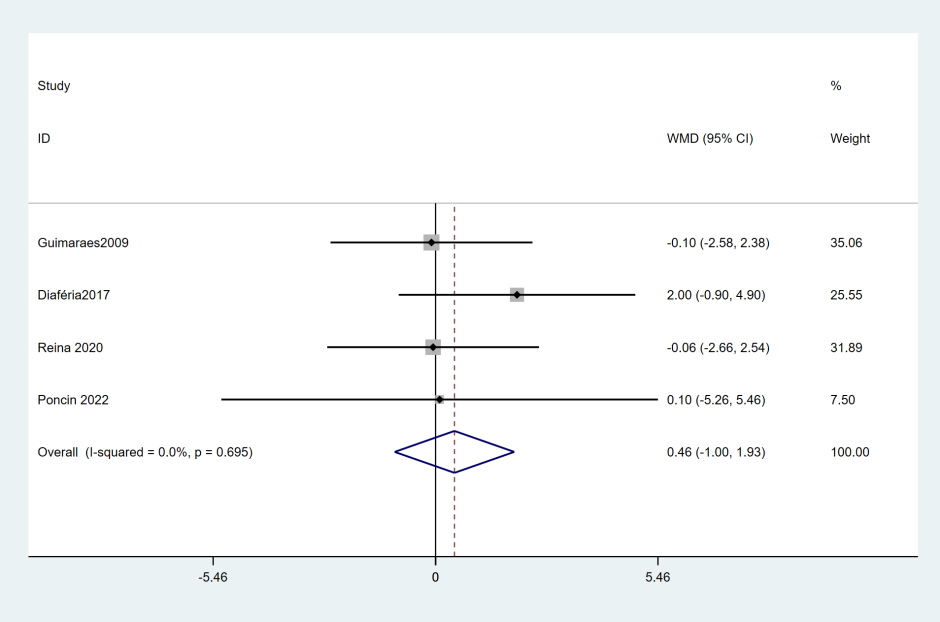
**

**Appendix 7 League table**

**7.1 Comparison of effect sizes for AHI**

| **ET** | — | — | — | — |
| --- | --- | --- | --- | --- |
| -1.09 (-6.34, 4.17) | **OMT** | — | — | — |
| -4.96 (-10.73, 0.82) | -3.87(-10.51, 2.77) | **WLD** | — | — |
| -8.11 (-15.86, -0.37) | -7.03(-15.20, 1.15) | -3.16(-11.92, 5.61) | **RMT** | — |
| -9.13 (-12.04, -6.21) | -8.04(-12.42, -3.66) | -4.17(-9.15, 0.81) | -1.01 (-8.21,6.18) | **CG** |

**7.2 Comparison of effect sizes for ESS**

| **OMT** | — | — | — | — |
| --- | --- | --- | --- | --- |
| -1.12 (-3.40,1.16) | **ET** | — | — | — |
| -1.39 (-3.73,0.95) | -0.27 (-2.95,2.42) | **RMT** | — | — |
| -3.00 (-6.02,0.02) | -1.88 (-5.06,1.30) | -1.61 (-4.95,1.73) | **WLD** | — |
| -4.00 (-5.45,-2.56) | -2.88 (-4.64,-1.12) | -2.61 (-4.64,-0.58) | -1.00 (-3.65,1.65) | **CG** |

**7.3 Comparison of effect sizes for PSQI**

| **RMT** | — | — | — |
| --- | --- | --- | --- |
| -0.25 (-1.92,1.41) | **ET** | — | — |
| -0.39 (-1.86,1.07) | -0.14 (-1.70,1.42) | **OMT** | — |
| -2.20 (-3.23,-1.16) | -1.94 (-3.25,-0.64) | -1.80 (-2.85,-0.76) | **CG** |

**7.4 Comparison of effect sizes for BMI**

| **WLD** | — | — | — | — |
| --- | --- | --- | --- | --- |
| -1.57 (-3.15,-0.00) | **ET** | — | — | — |
| -1.77 (-4.81,1.27) | -0.20 (-2.82,2.42) | **RMT** | — | — |
| -2.39 (-3.95,-0.84) | -0.82 (-1.04,-0.60) | -0.62 (-3.24,1.99) | **CG** | — |
| -2.86 (-5.00,-0.71) | -1.28 (-2.78,0.21) | -1.09 (-4.09,1.91) | -0.47 (-1.94,1.01) | **OMT** |

**Appendix 8 Cumulative ranking probability curves**

**8.1 Cumulative Ranking Probability Curves of AHI**


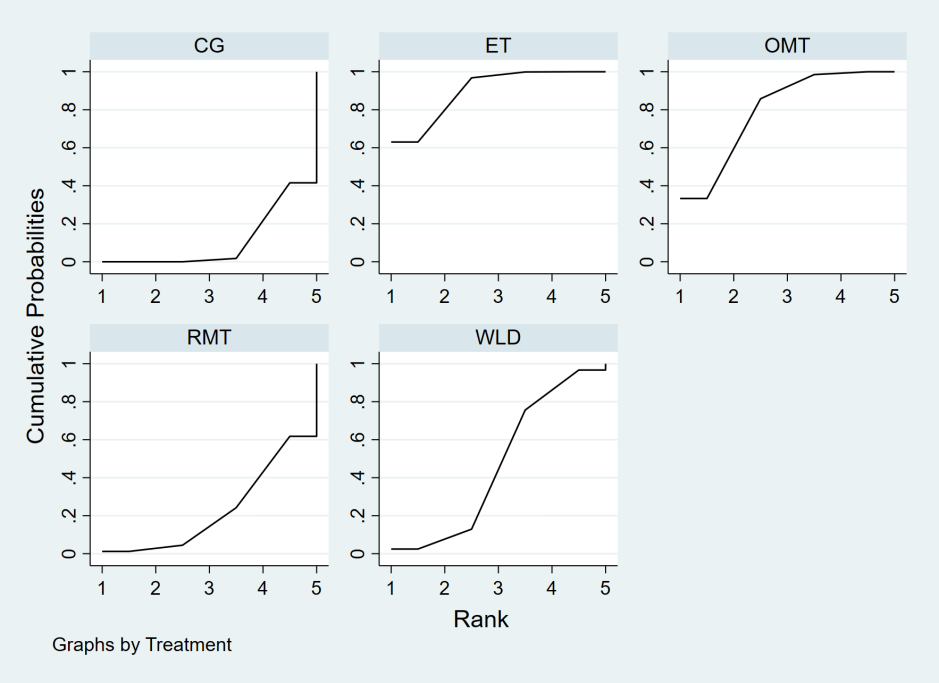


**8.2 Cumulative Ranking Probability Curves of ESS**


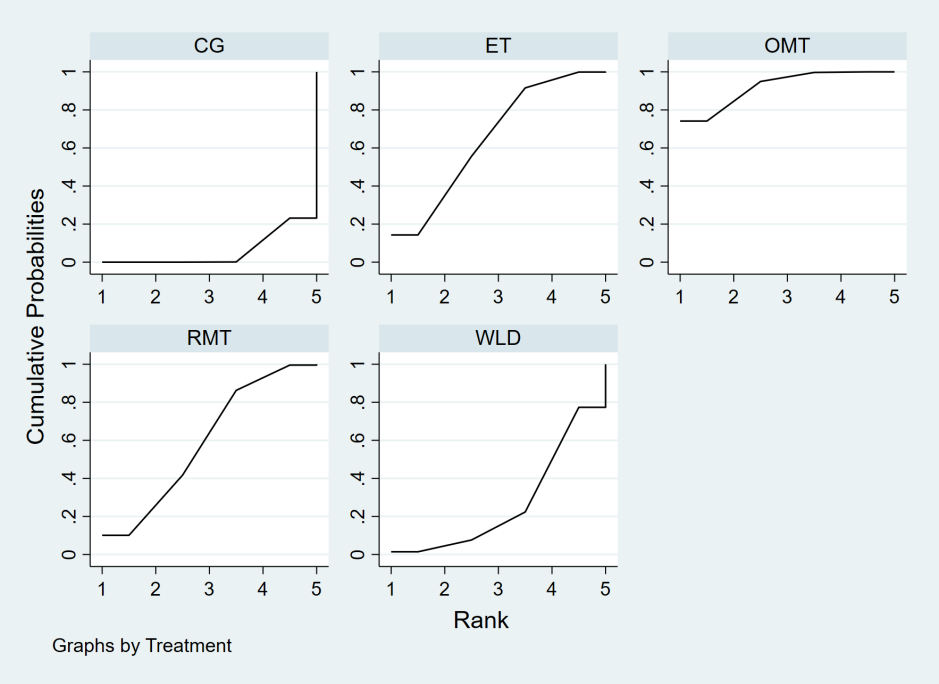


**8.3 Cumulative Ranking Probability Curves of PSQI**


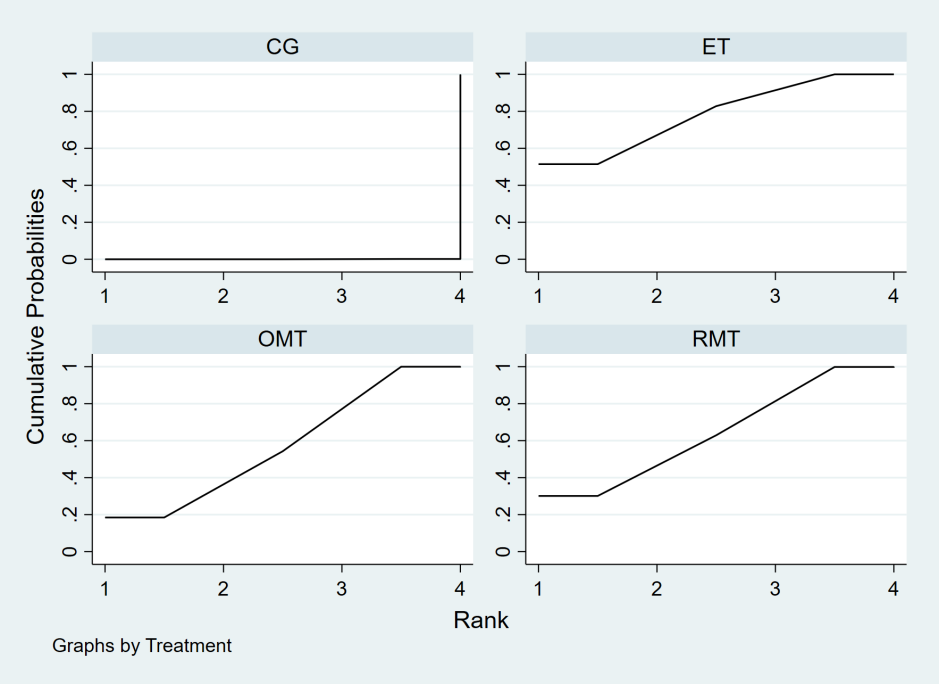


**8.4 Cumulative Ranking Probability Curves of BMI**


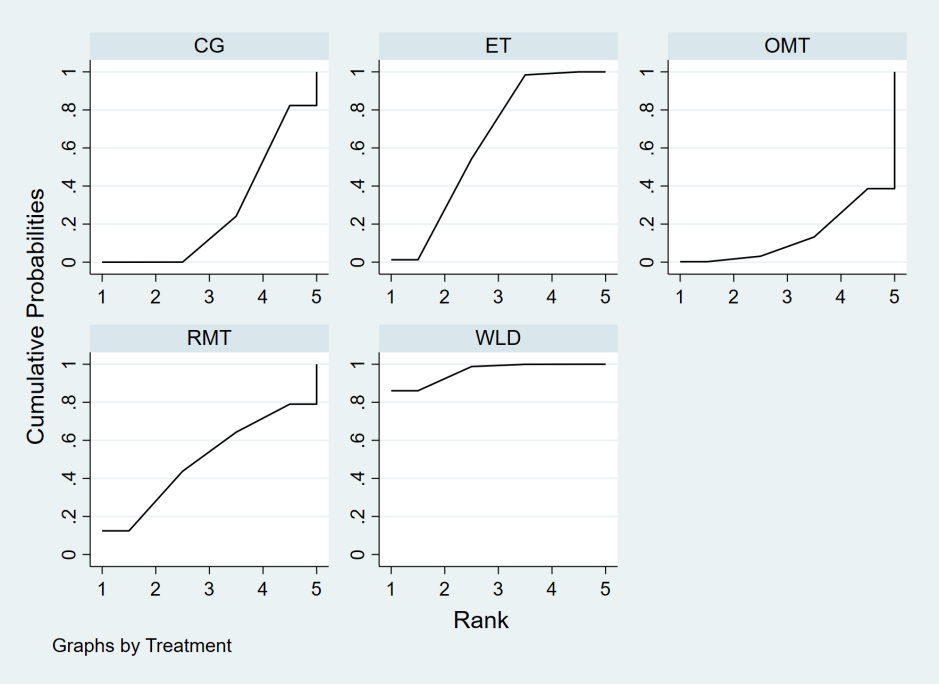


**Appendix 9 Subgroup analysis**

**9.1 Forest Plot of AHI**


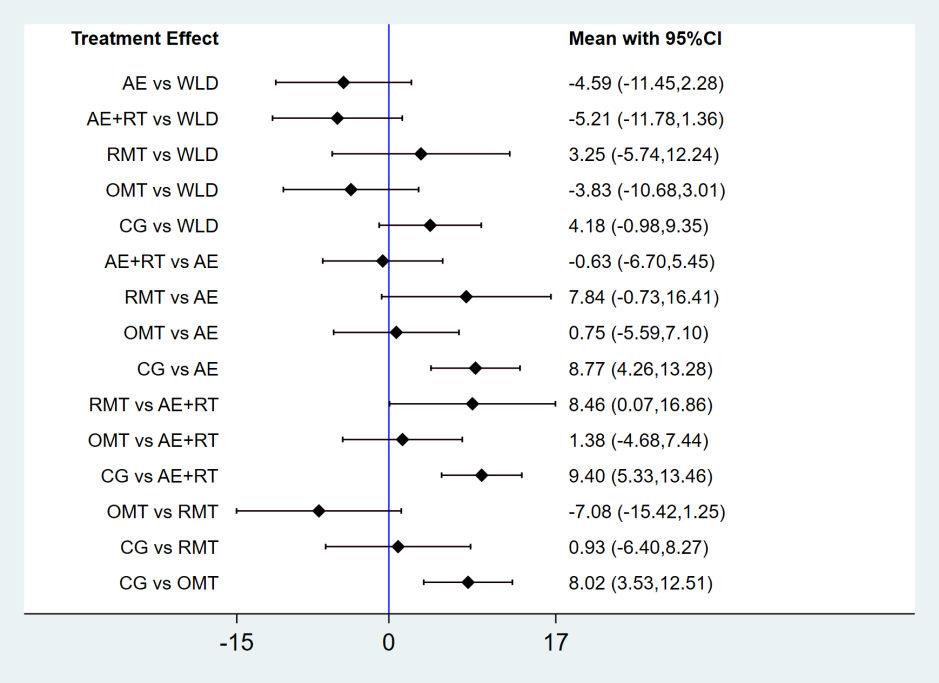


**9.2 Forest Plot of ESS**


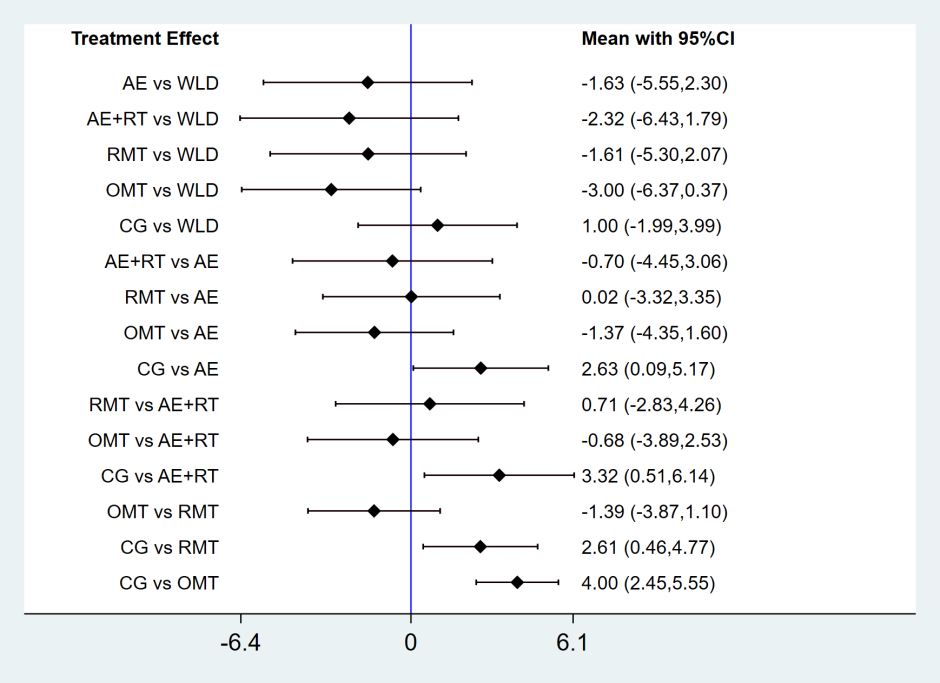


**9.3 Forest Plot of PSQI**


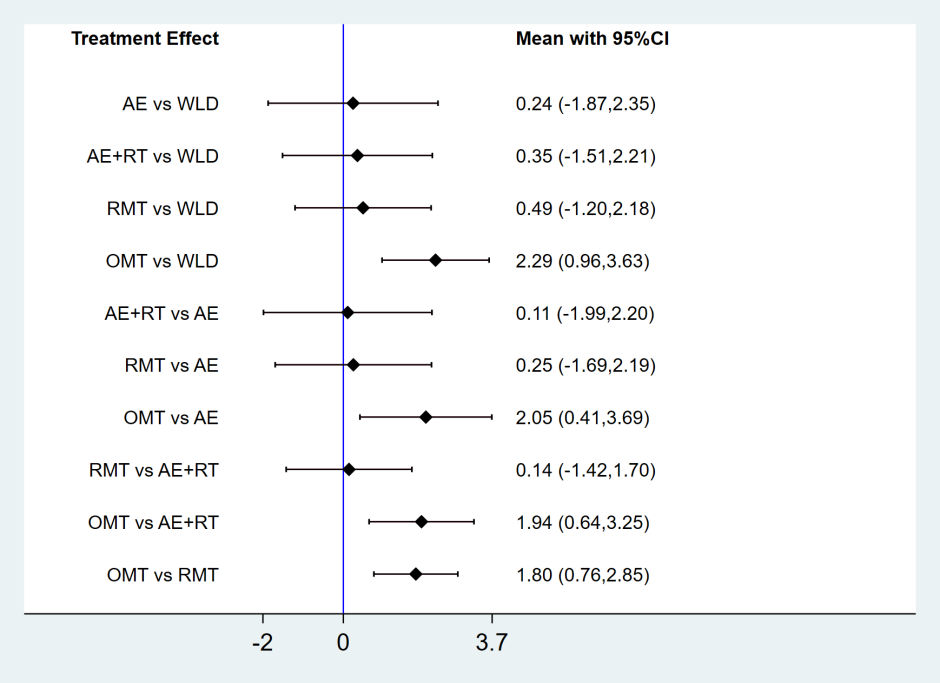


**9.4 Forest Plot of BMI**


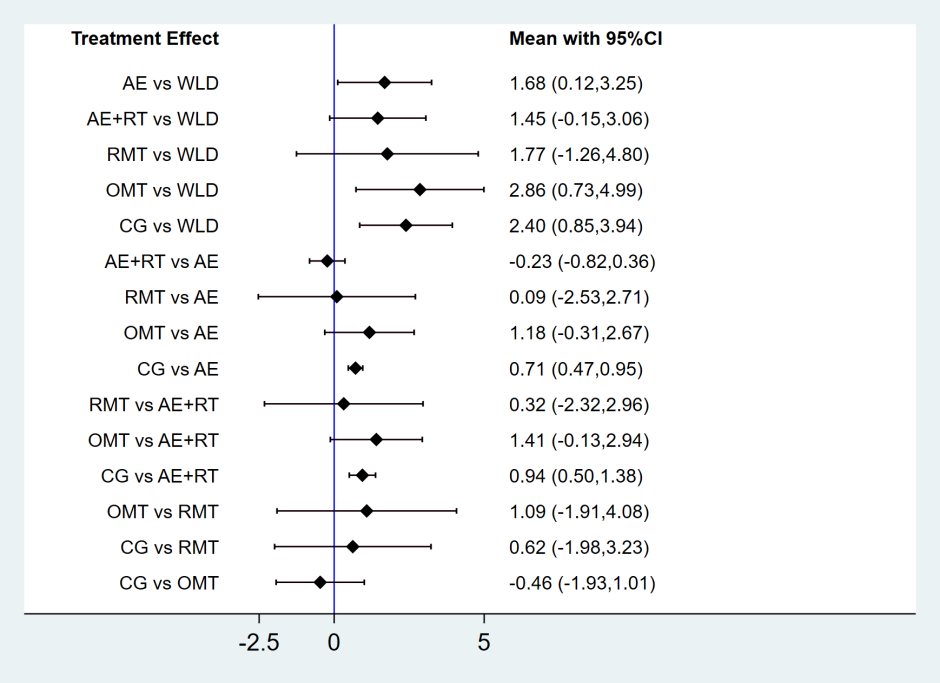


**9.5. Ranking table of SUCRA values.**

| Intervention | AHI | ESS | PSQI | BMI |
| --- | --- | --- | --- | --- |
| WLD | 39.8% | 26.2% | NA | 96.5% |
| AE | 77.6% | 57.1% | 72.7% | 54.7% |
| AE+RT | 83.6% | 69.9% | 63.4% | 67.2% |
| RMT | 19.3% | 55.4% | 60.1% | 47.9% |
| OMT | 70.9% | 85.5% | 53.5% | 12.1% |
| CG | 8.9% | 5.9% | 0.3% | 21.6% |

AE = exercise alone. RT= resistance training. NA = not available. The abbreviations used in both the figures and tables are consistent with the above definitions.

**Appendix 10 Sensitivity analysis.**

**10.1 Forest Plot of AHI**


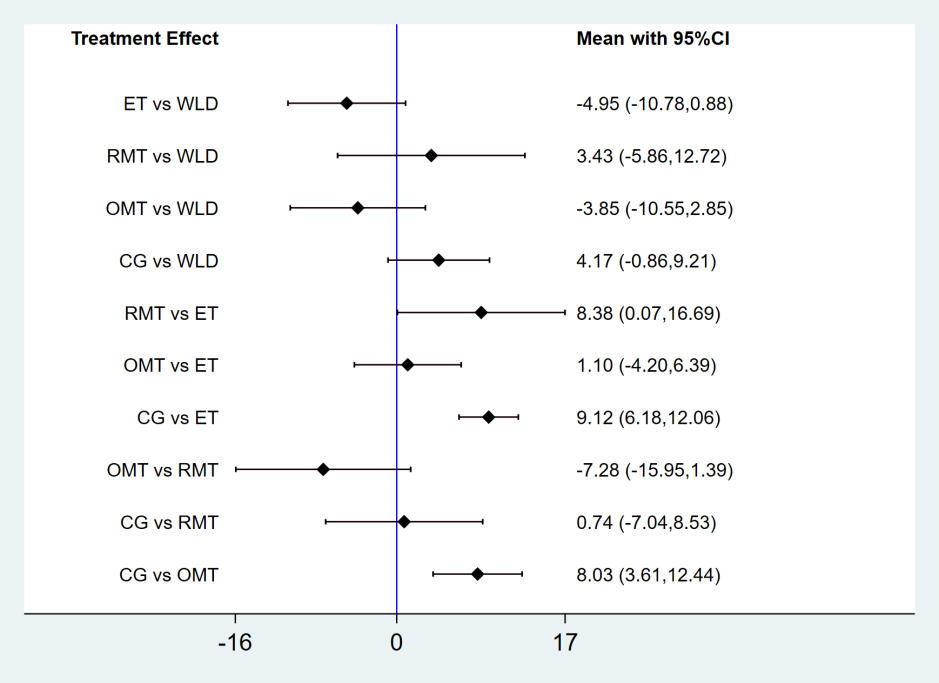


**10.2 Forest Plot of ESS**


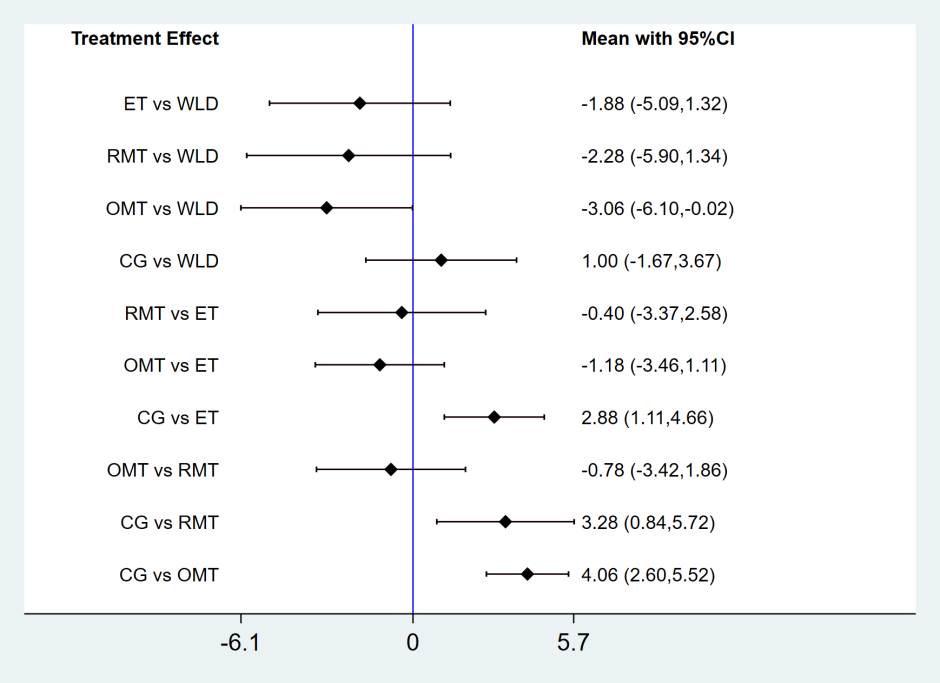


**10.3 Forest Plot of PSQI**


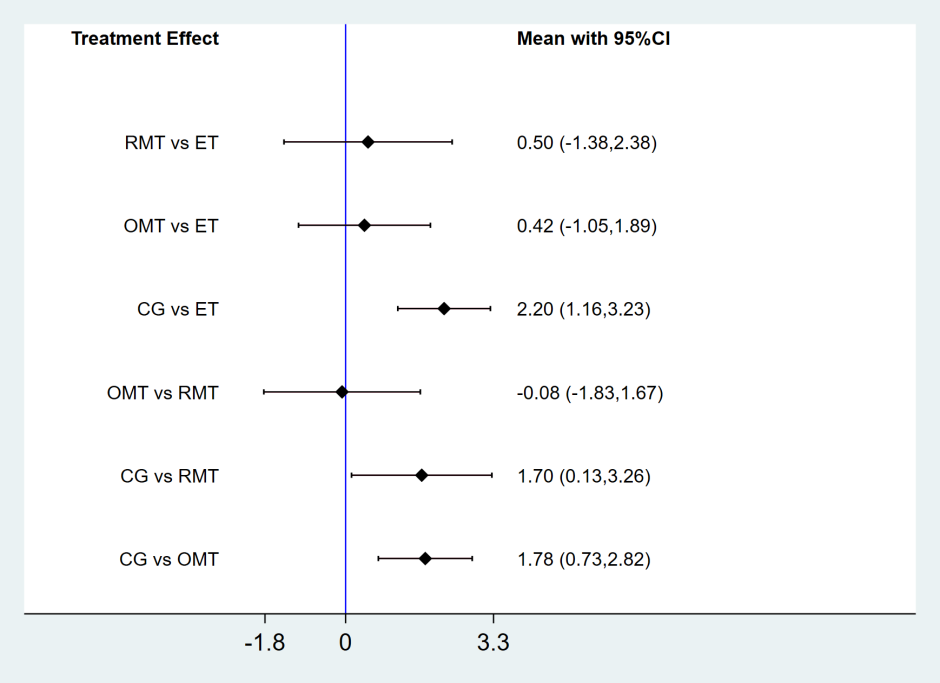


**10.4 Forest Plot of BMI**


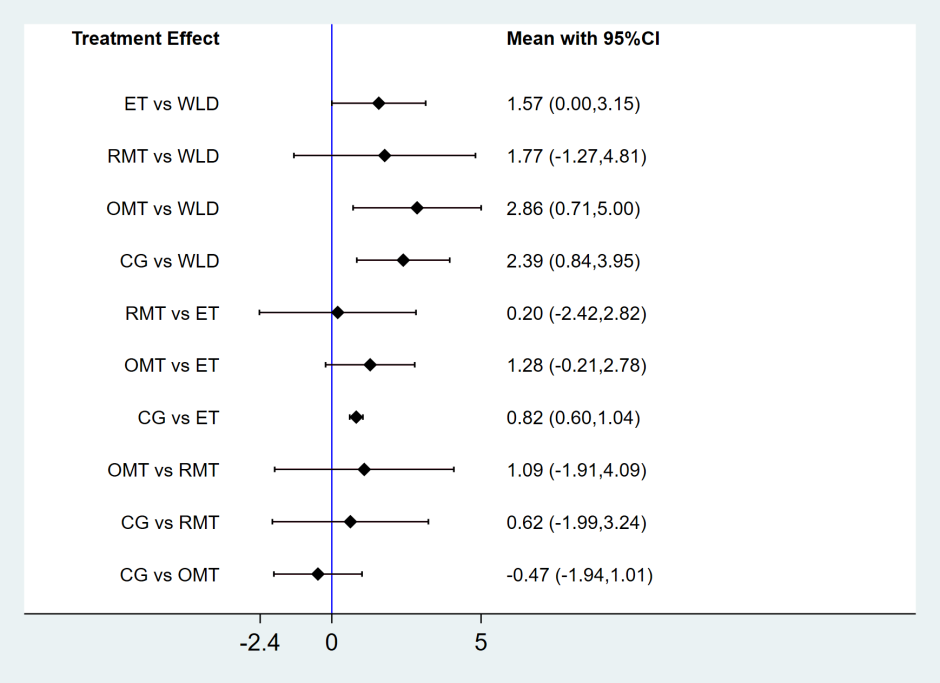


**10.5. Ranking table of SUCRA values.**

| Intervention | AHI | ESS | PSQI | BMI |
| --- | --- | --- | --- | --- |
| WLD | 46.8% | 26.0% | NA | 96.1% |
| ET | 89.9% | 60.4% | 80.7% | 63.6% |
| RMT | 21.1% | 69.5% | 57.7% | 50.3% |
| OMT | 79.1% | 88.3% | 60.9% | 13.7% |
| CG | 12.0% | 5.7% | 0.7% | 26.3% |

**Appendix 11 Egger's test**

| Outcome | Coef. | SE | 1. **\| t \|** | t | 95% CI |
| --- | --- | --- | --- | --- | --- |
| AHI | -1.302 | 0.677 | -1.93 | 0.066 | (-2.70, 0.09) |
| ESS | 1.536 | 0.930 | 1.65 | 0.120 | (-0.45, 3.52) |
| PSQI | 0.835 | 0.910 | 0.92 | 0.375 | (-1.13, 2.80) |
| BMI | -0.071 | 0.331 | -0.21 | 0.834 | (-0.78, 0.63) |
